# Non-coding RNA in neural function, disease, and aging

**DOI:** 10.3389/fgene.2015.00087

**Published:** 2015-03-09

**Authors:** Kirk Szafranski, Karan J. Abraham, Karim Mekhail

**Affiliations:** ^1^Department of Laboratory Medicine and Pathobiology, Faculty of Medicine, University of TorontoToronto, ON, Canada; ^2^Canada Research Chairs Program, Faculty of Medicine, University of TorontoToronto, ON, Canada

**Keywords:** miRNA, lncRNA, R-loops, TDP-43, FUS, ATXN2, SETX, neurodegeneration

## Abstract

Declining brain and neurobiological function is arguably one of the most common features of human aging. The study of conserved aging processes as well as the characterization of various neurodegenerative diseases using different genetic models such as yeast, fly, mouse, and human systems is uncovering links to non-coding RNAs. These links implicate a variety of RNA-regulatory processes, including microRNA function, paraspeckle formation, RNA–DNA hybrid regulation, nucleolar RNAs and toxic RNA clearance, amongst others. Here we highlight these connections and reveal over-arching themes or questions related to recently appreciated roles of non-coding RNA in neural function and dysfunction across lifespan.

## Introduction

Neurodegenerative diseases are a group of debilitating neurological disorders typically associated with old age (Eacker et al., 2009). These conditions are characterized by the progressive loss of neurons within one or more regions of the CNS (Eacker et al., 2009). Most neurodegenerative diseases are sporadic in origin, meaning that they arise in patients with no apparent family history or known environmental cause (Robberecht and Philips, 2013). While the biology of sporadic disease remains mysterious, several decades of research on various inherited forms have led to remarkable advances in our understanding of disease genetics. Systematic efforts over the last few decades have led to the identification of a number of causal mutations and key genes (Pulst et al., 1996; Neumann et al., 2006; Kabashi et al., 2008). Building on these findings, many studies have attempted to delineate the precise pathological processes arising from such genetic anomalies. A number of disease mechanisms have been proposed including toxicity induced by misfolded or aberrantly translated proteins, and the mislocalization of important cellular factors (Eacker et al., 2009). Despite encouraging progress, many aspects of these disorders are poorly understood and sadly, many devastating conditions remain untreatable. Furthermore, diseases for which symptom-relieving treatments are available are often difficult to diagnose. As a whole, neurodegenerative disease represents a major source of morbidity and mortality worldwide and is justifiably garnering the attention of both the scientific community and society at large.

Neurodegenerative diseases are associated with a diverse spectrum of clinical presentations, brain pathologies, and health consequences (Eacker et al., 2009). They include relatively well-known conditions like AD, FTLD, PD, and ALS. Additionally, a host of less prevalent disorders including various SCAs and AOA2 also belong to this class of diseases. Conditions, like AD and FTLD are primarily cognitive disorders and represent the two leading causes of dementia worldwide (Mattson, 2004). They are caused by progressive loss of brain regions responsible for reasoning, cognition, and memory. In contrast, ALS and PD are chiefly disorders of motor function, caused by the preferential loss of motor neurons and dopamine-producing neurons in the brain, respectively (Hardiman et al., 2011; Wirdefeldt et al., 2011). Although some conditions can be contained by pharmacotherapy and/or surgery, the majority of neurodegenerative diseases remain untreatable. Furthermore, neurodegenerative diseases represent an increasing fiscal burden in healthcare systems attempting to cope with aging populations. Thus, there is an urgent need to develop approaches that prevent, stall, or cure neurodegenerative disease. Ultimately, this process will hinge on understanding the precise molecular and cellular mechanisms that lead to the premature dysfunction or death of neurons.

**FIGURE 1:**
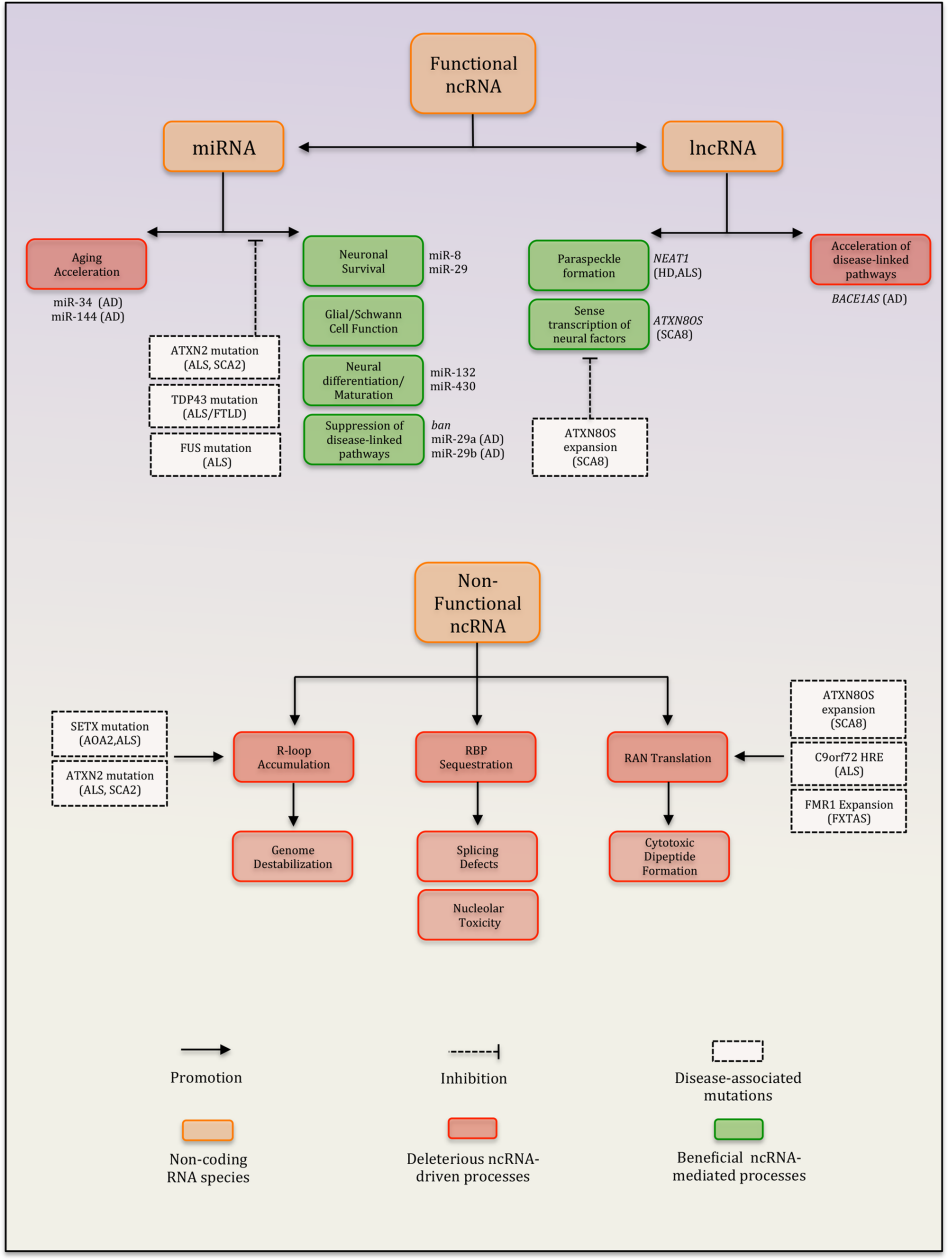
Summary of ncRNA-dependent processes implicated in neurodegenerative disease. Diseases highlighted: AD, Alzheimer’s disease; HD, Huntington’s disease; SCA8, spinocerebellar ataxia type 8; SCA2, spinocerebellar ataxia type 2; ALS, amyotrophic lateral sclerosis; AOA2, ataxia with oculomotor apraxia type 2; FXTAS, fragile X-associated tremor/ataxia syndrome; and FTLD, frontotemporal dementia.

Basic research on potential disease mechanisms has benefited greatly from studies using model organisms and/or novel experimental systems. Insights from such studies are providing mounting evidence that ncRNAs and ncRNA-regulatory processes are important players in the pathogenesis of neurodegenerative disease (Eacker et al., 2009; Esteller, 2011; Abe and Bonini, 2013). ncRNAs represent a functionally and structurally diverse class of RNA species that participate in a wide range of basic cellular processes including protein translation, mRNA splicing, chromatin organization, and the regulation of gene expression (Esteller, 2011). Several classes of ncRNAs (e.g., miRNAs, rRNAs, tRNAs, and many lncRNAs) fulfill discrete functions within cells. However, it is becoming clear that a large proportion of the cellular transcript pool is comprised of ncRNAs that lack obvious function (Djebali et al., 2012; Palazzo and Gregory, 2014). These entities are thought to derive primarily from noisy transcription at intergenic sequences and are generally degraded rapidly within the nucleus (Djebali et al., 2012; Palazzo and Gregory, 2014). However, recent studies suggest that under certain conditions, these ncRNAs can trigger processes that are toxic to cells (**Figure 1**). These processes include the sequestration of crucial RNA-binding proteins as well as the accumulation of genome-destabilizing R-loops, which are structures that can form when nascent RNA stably hybridizes with DNA (Aguilera and Garcia-Muse, 2012; Haeusler et al., 2014; Salvi et al., 2014). Accordingly, cells have evolved a number of mechanisms to constrain both the amount and the ability of transcripts to engage in these detrimental processes (Palazzo and Gregory, 2014; Salvi et al., 2014). In this review, we focus exclusively on ncRNAs and make the broad distinction between ncRNAs that are either functional or lack a discrete role but nevertheless represent important determinants of overall cellular function. Specifically, we discuss the links between neurodegenerative disease and two major classes of functional ncRNAs, namely miRNAs and a group of lncRNAs. Additionally, we explore emerging evidence that deleterious ncRNA-driven processes such as R-loop formation and toxic RNA accumulation represent neurotoxic mechanisms in brain aging and neurodegeneration.

## Roles of miRNAs, lncRNAs, and other ncRNAs in Neural Function and Aging

### Introduction to miRNAs: Biogenesis and Regulatory Mechanisms

**FIGURE 2:**
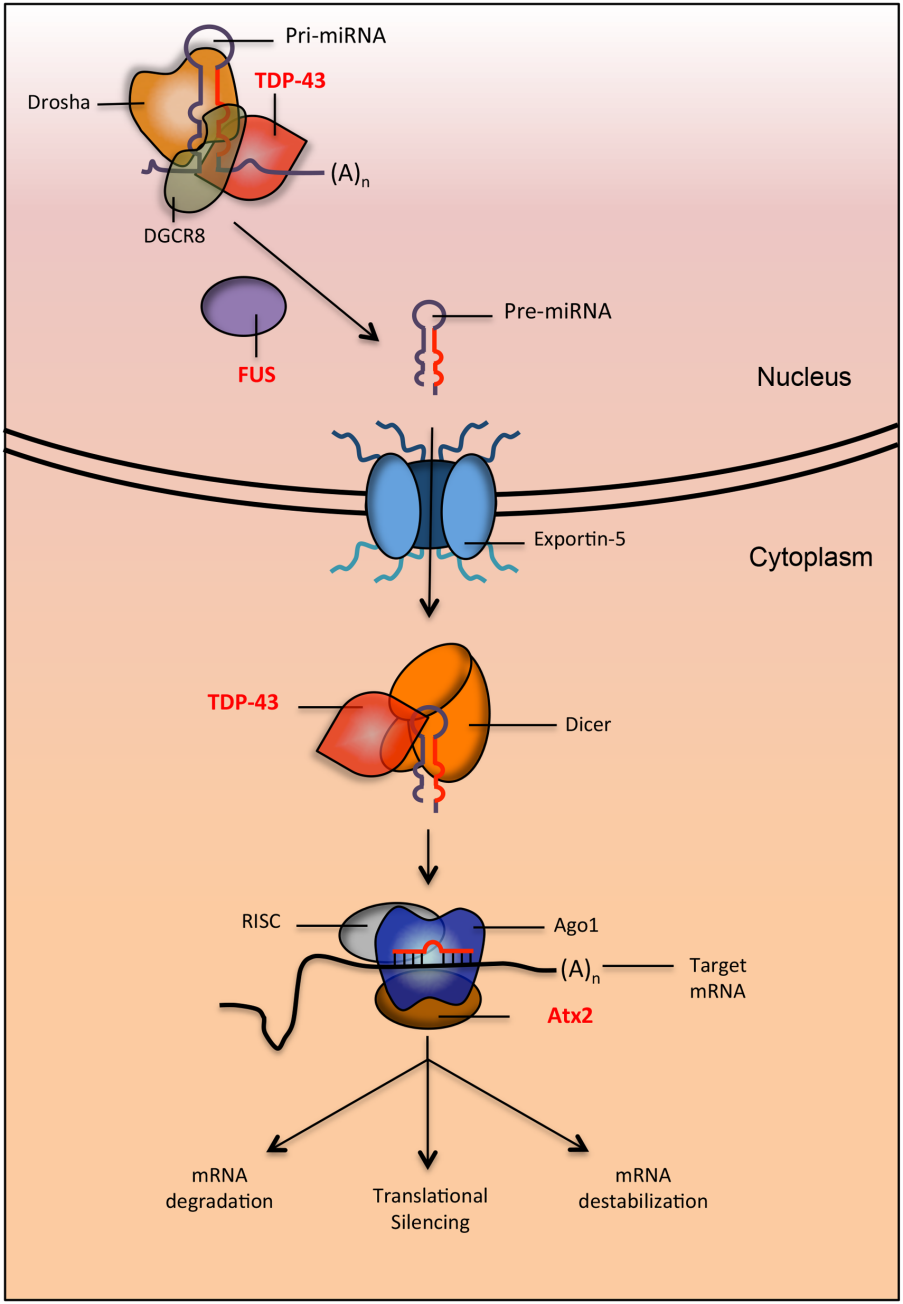
Proteins implicated in neurodegenerative diseases are involved in multiple steps of the miRNA biogenesis pathway. TDP-43 and FUS (implicated in ALS and FTLD) interact with Drosha (directly and indirectly respectively) to promote the processing of pri-miRNAs into pre-miRNA. After export, TDP-43 also interacts with dicer to process pre-miRNA into mature miRNA. When miRNA is loaded onto the RISC to be targeted for silencing, it interacts with Ago1. In *Drosophila*, it was shown that the protein Atx2 (the ortholog of ATXN2, a protein implicated in ALS and SCA2) interacts with Ago1 to promote proper targeting of miRNAs to their mRNA.

microRNAs are an abundant class of short ncRNAs that regulate a variety of cellular processes through the post-transcriptional repression of gene expression (He and Hannon, 2004). Mature miRNAs are generated following a series of well-orchestrated biochemical events that begin in the nucleus and culminate in the cytoplasm (**Figure 2**; Lee et al., 2002). These steps include the nuclear processing of primary miRNA transcripts (pri-miRNAs) into precursor miRNAs (pre-miRNAs) by the DGCR8/Drosha complex, the cytoplasmic processing of pre-miRNAs into imperfectly paired miRNA duplexes by Dicer, and the preferential incorporation of one strand (the “guide" miRNA strand) onto the RISC (Bartel, 2004). Mature miRNAs complexed to RISC (miRISC) are targeted to mRNAs containing sequences that are complementary to the miRNA “seed" (nucleotides 2–7 of a mature miRNA). Ultimately, the repression of mRNAs occurs via their degradation, destabilization, translational silencing, or combinations thereof. Regardless of the specific mode of repression, the final outcome typically consists of decreased target protein levels and a subsequent biological effect reflecting the cellular function of the targeted transcript.

microRNAs have been increasingly linked to processes associated with brain aging, declining brain function, and neurodegenerative diseases (Eacker et al., 2009; Abe and Bonini, 2013). Indeed, several studies have reported widespread changes in miRNA expression, while others have identified specific miRNAs as strong correlates of brain aging and/or specific disease states (Li et al., 2011a; Persengiev et al., 2011; Inukai et al., 2012; Sheinerman et al., 2013). Future developments may allow these correlations to be used as powerful diagnostic markers of neurodegenerative diseases. In this section, we discuss the involvement of miRNAs in regulating diverse aspects of neural function based on insights gained from various experimental and genetic model systems. Furthermore, we highlight both direct and indirect evidence for deregulation of miRNA pathways in neurodegenerative disease.

### miRNAs as Mediators of Brain Development and Neuronal Differentiation

A crude but effective approach to assess the overall role of miRNAs in the brain is to disable the miRNA biogenesis pathway (Eacker et al., 2009). This can be accomplished by the conditional deletion of Dicer, which results in a failure to generate mature dicer-dependent miRNAs. It is important to note that there have been reports of a relatively small number of dicer-independent miRNAs, which would not be affected by this strategy (Cheloufi et al., 2010; Ha and Kim, 2014). This strategy applied to model organisms has nevertheless helped unearth roles for miRNAs in developmental processes of the CNS including tissue morphogenesis and neuronal differentiation. In zebrafish, the loss of maternal and zygotic Dicer manifests in abnormal organogenesis. A striking component of this phenotype includes severe defects in neurulation and neural differentiation, which leads to failed brain morphogenesis (Giraldez et al., 2005). Several studies in mice have investigated the developmental consequences of disabling miRNA biogenesis in stem or neural precursor populations. One study used a Wnt1-cre promoter to conditionally delete Dicer in neural crest cells and select regions of the brain (Huang et al., 2010). This causes malformed development of the cerebellum and midbrain, and impaired differentiation of neural crest and dopaminergic cells. Similarly, the inactivation of Dicer in olfactory and post-mitotic motor progenitors leads to a failure to generate olfactory and post-mitotic motor neurons, respectively (Choi et al., 2008; Chen and Wichterle, 2012). The importance of miRNAs in neuronal differentiation is also supported by studies employing cell culture models of differentiation. For instance, the inactivation of miRNA biogenesis during the final stages of differentiation of DNs derived from murine embryonic stem cells abolishes terminal differentiation in culture (Kim et al., 2007b). Taken together, these studies identify miRNAs as critical players in fundamental brain development processes such as neuronal differentiation. Thus, disruption of miRNA biogenesis can compromise neurodevelopmental and neural differentiation processes that are known to occur at various stages of life.

### Roles for miRNAs in Neuronal Longevity and Survival

Premature death of neurons is a major characteristic of neurodegenerative diseases, and the root cause of many health complications that arise during the course of illness. Mice have been used extensively to address a wide variety of questions relating to neuronal lifespan, including the need for a functional miRNA pathway. To determine the role of miRNAs in the maintenance of neuronal lifespan *in vivo*, several studies have characterized the neurobiological phenotypes caused by the deletion of Dicer in discrete populations of terminally differentiated cells. In many cases, the absence of a functional miRNA pathway leads to the loss of neurons via both cell autonomous and non-cell autonomous mechanisms. The inactivation of Dicer in post-mitotic murine midbrain neurons for instance, manifests in a drastic reduction in number and axonal projection of DNs residing in the midbrain as evidenced by decreased expression of DN markers on immunohistochemical brain sections (Kim et al., 2007b). This mirrors the pathological changes observed in human PD. Moreover, these degenerative changes are accompanied by marked reductions in locomotion and mobility reminiscent of bradykinesia (i.e., slowing of movement) seen during early stages of PD (Kim et al., 2007b). Region-specific neurodegeneration is observed in cerebellar Purkinje cells (PCs) devoid of Dicer (Schaefer et al., 2007). Interestingly, 10-weeks-old wild-type and Dicer-deficient mice display no differences in morphology or electrophysiological functions of PCs. Between 13 and 17 weeks, Dicer-deficient mice show profound PC apoptosis as revealed by electron microscopy, immunohistochemistry, and TUNEL staining. Furthermore, cerebellar degeneration coincides with onset of ataxia and a mild tremor that ultimately deteriorates to severely impaired coordination and motor function. Similarly, conditional deletion of Dicer in the spinal motor neurons results in deteriorating motor function from week 7 onward and histological changes in muscle that are pathognomonic for denervation-induced muscular atrophy (Schaefer et al., 2007; Damiani et al., 2008; Haramati et al., 2010). Indeed, spinal cord sections from these mice display reduced numbers of lower motor neurons and immunohistochemical evidence of reactive astrocytosis, which is a marker of neurotoxicity (Schaefer et al., 2007; Damiani et al., 2008; Haramati et al., 2010). Thus, the absence of a functional miRNA pathway diminishes survival and recapitulates various clinical and pathological features of human neurodegenerative disease. This highlights the vital role of the miRNA pathway in maintaining normal neuronal lifespan and points to the potential deregulation of this pathway as a driver or contributor to neurodegeneration.

Neurons rely on many supporting cell types (astrocytes, oligodendrocytes, Schwann cells, etc.) that co-exist in the nervous system. Several studies have shown that defective miRNA biogenesis in these cells abrogates critical homeostatic functions and lead to the death of neurons via non-cell autonomous mechanisms. The loss of Dicer in astroglial cells results in impaired neurotransmitter uptake and increased oxidative stress (Tao et al., 2011). Disruption of these critical functions was associated with ataxia at post-natal week 7, followed by prolonged seizures, severe locomotor dysfunction and eventually premature organismal death. Immunohistochemistry in combination with electron microscopy and TUNEL staining in cerebellar sections revealed that these changes were precipitated by apoptosis and axonal degeneration of mature cerebellar granule cells and PCs, respectively. Similarly, the loss of Dicer in Schwann cells and oligodendrocytes triggers inflammatory and oxidative cascades in the brain leading to neuronal injury and degeneration (Shin et al., 2009; Bremer et al., 2010). Thus, in the setting of neurodegenerative disease, loss of key homeostatic functions downstream of miRNA biogenesis can induce non-cell autonomous neuronal degeneration (**Figure 1**).

It is important to note that miRNAs are required for many aspects of neuronal structure and function, not only their long-term survival. In several studies, disabling miRNA biogenesis manifests in complex phenotypes in which degeneration is preceded by structural and/or functional abnormalities. For instance, Dicer inactivation in retinal cells results in the formation of abnormal rosette structures composed of photoreceptors prior to degeneration (Damiani et al., 2008). Similarly, the deletion of Dicer in cortical and hippocampal neurons is associated with abnormalities in cellular morphology, tissue architecture, and axonal pathfinding followed by profound degeneration within these regions (Davis et al., 2008). Thus, miRNA dysfunction may trigger overt neuronal degeneration as well as more subtle deficits in the setting of neurodegenerative disease.

### Regulation of Neurodegenerative Disease-Associated Pathways by miRNAs

While previously discussed studies tested the need for global miRNA function in the brain, many other studies have focused on the relevance of specific miRNAs or miRNA families in regulating either neuroprotective or neurotoxic processes. One family of miRNAs that has emerged as putatively protective is the miR-29 family (**Figure 1**). Several studies employing mouse models and human cell lines have examined the involvement of miR-29 in neurodegenerative diseases. In agreement with a critical role in the brain, miRNA profiling studies have reported that miR-29a is expressed abundantly in neurons in the adult mouse brain, while miR-29b expression increases in various compartments of the maturing mouse CNS (Kole et al., 2011; Jovicic et al., 2013). Consistent with a role in neuroprotection, diminished levels of miR-29 family members have been reported in both patients or mouse models of AD, HD, and various subtypes of SCA (Lee et al., 2011; Wang et al., 2011b). The notion that these patterns may represent disease-promoting mechanisms rather than just correlational markers stems from the observation that perturbing miR-29 function is associated with compromised neuronal survival. For instance, knocking down miR-29b in the mouse brain using LNA-based antagomirs induces severe cerebellar and hippocampal degeneration, which manifests in ataxia and eventually leads to death (Roshan et al., 2014). This phenotype appears to be mediated in part by the de-repression of VDAC1, an apoptotic effector protein and miR-29b target. Reduced miR-29b levels are also associated with increased susceptibility to neuronal apoptosis in mouse models of acute ischemic stroke (Khanna et al., 2013). In this study, partial phenotypic rescue was achieved by the restoration of miR-29b expression. Lastly, the miR-29 family may also promote neuroprotection via the direct suppression of disease-linked pathways (**Figure 1**). In the setting of AD, the expression of miR-29a/b-1 is inversely associated with BACE-1 levels (Hebert et al., 2008). BACE-1 is an enzyme that can cleave amyloid precursor protein (APP) into amyloid beta (Aβ) peptides, the chief component of Aβ plaques found in the brains of AD patients (O’Brien and Wong, 2011). In human cell lines modeling Aβ plaque formation, miR-29a/b-1 can directly suppress the expression of BACE-1 via direct seed-3′ UTR interactions and reduce Aβ plaque formation *in vitro* (Hebert et al., 2008). These results suggest that in disease states like AD, downregulation of miR-29a/b-1 may promote disease susceptibility and/or progression in patients by precipitating Aβ plaque formation. Overall, functional studies focusing on miR-29 align well with its inverse association with neurodegenerative disease and point to a conserved role for this miRNA family in neuroprotection.

Examples of protective miRNAs that can directly target disease-associated proteins also exist in *Drosophila melanogaster*. For instance, the *ban* miRNA potently suppresses neurodegeneration induced by polyglutamine Ataxin-3 and tau, pathogenic proteins that are causally linked to SCA3 and AD, respectively (Bilen et al., 2006). Similarly,* Drosophila* miR-8 functions as a neuroprotective factor by targeting atrophin, a family of proteins linked to the neurodegenerative disease dentatorubral-pallidoluysian atrophy (DRPLA; **Figure 1**; Ross et al., 1997; Karres et al., 2007). These results raise the possibility that analogous pathways exist in humans, and future studies that explore this further may furnish novel opportunities for miRNA-based therapeutic targeting of disease proteins.

While some miRNAs are clearly protective, others may promote brain aging and/or initiate neurotoxic processes that contribute to specific neurodegenerative diseases (**Table 1**). For instance, miR-144 is a strong positive correlate of aged brains in humans, chimpanzees, and rhesus macaques (Persengiev et al., 2011). miR-144 is also enriched in post-mortem tissue from SCA1 and AD patients. Importantly, the ATXN1 protein (encoded by the gene mutated in SCA1) is a known target of miR-144. Thus, age-associated miR-144 may contribute to declining brain function in both normal and disease states via the downregulation of longevity/protective factors (**Figure 1**; Persengiev et al., 2011). Similarly, studies in both *D. melanogaster* and *Caenorhabditis elegans* point to the miR-34 family as another important determinant of brain aging. The miR-34 family is a highly conserved set of brain-enriched miRNAs (Bak et al., 2008). Intriguingly, correlative studies in mice and humans seem to point to a potential role for miR-34 in AD. Brain tissue derived from a mouse model of AD and in post-mortem tissue from AD patients are enriched for miR-34a (Wang et al., 2009). Interestingly, the pro-survival protein Bcl2 is a known miR-34a target. Furthermore, the expression of another known target of miR-34a, namely the anti-aging factor SIRT1, correlates inversely with age-associated increases in brain and circulating levels of that miRNA (Li et al., 2011b). SIRT1 knockdown accelerates neurodegeneration due to mutant HTT in mouse models of HD, while overexpression rescues phenotypes (Jiang et al., 2012). Moreover, SIRT1 promotes neuronal survival and suppresses neurodegeneration in a mouse model of AD and ALS (Kim et al., 2007a). Similarly, several reports are consistent with the notion that SIRT1 is neuroprotective (Araki et al., 2004; Chen et al., 2008a; Shindler et al., 2010; Huang et al., 2011; Graff et al., 2013). However, overt neurodegenerative defects have not yet been clearly documented in SIRT1-null mice, which nonetheless exhibit neural migration defects (Di Sante et al., 2015). Thus, SIRT1 may be simply neuroprotective or compensatory processes mediated by other sirtuin proteins may be involved. Consistent with the latter rationale, Sirt6 modulates neural chromatin structure and gene activity (Schwer et al., 2010). Taken together, while the precise links between neurodegeneration and miR-34a remain unclear, repression of protective factors such as Bcl2 and SIRT1 by the latter miRNA may point to two non-mutually exclusive possibilities.

**Table 1:** List of the various transcripts and proteins discussed in this review as well as their associated disease and proposed pathogenic mechanism.

Transcript/protein	Organism studied	Diseases implicated	Proposed mechanism	Reference
*ban*	Fly	SCA3, AD	Suppresses neurodegeneration by inhibiting tau and Ataxin-3	Bilen et al. (2006)
*miR-8*	Fly	DRPLA	Represses atrophin	Ross et al. (1997); Karres et al. (2007)
*miR-29*	Mouse, human	AD, HD, SCA	Represses VDAC1, BACE-1	Hebert et al. (2008); Roshan et al. (2014)
*miR-144*	Human, chimpanzee, macaque	Aging, SCA1	Represses ATXN1, other candidates	Persengiev et al. (2011)
*miR-34*	Fly, worm, mouse, human	AD	Represses protective factors Bcl2 and SIRT1	Wang et al. (2009); Li et al. (2011b)
TDP-43	Mouse, human	ALS, FTLD	Interacts with Drosha to facilitate processing of miRNAs	Kawahara and Mieda-Sato (2012)
*miR-132*	Human	ALS	Neural outgrowth factor regulated by TDP-43 and FUS	Kawahara and Mieda-Sato (2012); Morlando et al. (2012)
FUS/TLS	Human	ALS, FTLD	Indirect Drosha interactions regulates various miRNAs	Morlando et al. (2012)
ATX2	Fly	ALS, SCA2, PSP	Interactions with RISC components regulates long-term habituation	McCann et al. (2011)
DGCR8	Human	Prader–Willi syndrome	Microprocessor, can process miRNAs and snoRNAs	Macias et al. (2012)
*n-Tr20*	Mouse	N/A. Neural cell death observed with mutation	tRNA isodecoder necessary for the production of GTPBP2	Ishimura et al. (2014)
CLP1	Human	PCH 10	Necessary for maturation of tRNAs	Schaffer et al. (2014)
*BACE1AS*	Human	AD	Stabilization of *BACE1* which ultimately results in amyloid beta accumulation	Faghihi et al. (2008)
*ATXN8OS*	Human	SCA8	May reduce the amount of KLHL1, may produce toxic dipeptides or sequester RNA binding proteins	Chen et al. (2008b); Zu et al. (2011); Tsoi et al. (2012)
*UCHL1-AS*	Mouse	AD, PD	Regulates translation of neuroprotective protein UCHL1	Carrieri et al. (2012)
*NEAT1*	Mouse, human	ALS, HD, aging	Produces paraspeckle bodies which storage RNAs, potentially for release during stress	Prasanth et al. (2005); Johnson (2012); Abdelmohsen et al. (2013); Nishimoto et al. (2013)
*C9ORF72*	Fly, human	ALS	Abortive transcripts sequester RNA binding proteins and produce toxic dipeptides	Haeusler et al. (2014); Mizielinska et al. (2014)
SETX	Human, yeast	AOA2, ALS	RNA–DNA helicase prevents R-loop formation	Skourti-Stathaki et al. (2011); Yuce and West (2013)
Pbp1	Yeast	ALS, SCA2, PSP	Interacts with RNAs to prevent RNA–DNA hybrids, maintains genomic stability, and cellular lifespan	Salvi et al. (2014)

*Transcripts are indicated by italicized text, while proteins are underlined*.

### miRNA Dysfunction Downstream of Disease-Associated Proteins

Several neurodegenerative diseases are associated with defects in key RNA-binding proteins including TDP-43, ATXN2, and FUS. TDP-43 and FUS have roles in the regulation of coding RNAs which we do not discuss here and instead refer readers to several excellent reviews about TDP-43 and FUS function (Lagier-Tourenne and Cleveland, 2009; Baloh, 2012). In this section, we explore emerging evidence linking these disease-associated proteins with putative miRNA-regulatory functions and suggest how disruption of these roles may trigger pathological cascades in neurodegenerative diseases.

Tar-DNA binding protein-43 is a ubiquitously expressed RNA-binding protein strongly linked to several neurodegenerative diseases. Mutation of *TARDBP* (encodes TDP-43) is a relatively rare cause of familial ALS (Kabashi et al., 2008). Moreover, TDP-43 protein is frequently mislocalized to ubiquitinated inclusion bodies in ALS/FTLD, pointing to a role in disease pathogenesis even in the absence of *TARDBP* mutations (Neumann et al., 2006). In healthy cells, TDP-43 is primarily nuclear and it fulfills diverse roles in the regulation of mRNA transcription, alternative splicing, and ncRNA stability (Robberecht and Philips, 2013). However, it is becoming increasingly apparent that TDP-43 may additionally act as a regulator of miRNA biogenesis and function. Indeed, TDP-43 has been shown to physically interact with Drosha and Dicer in the nucleus and cytoplasm respectively, and these interactions are required for the normal processing of several miRNAs (Kawahara and Mieda-Sato, 2012). Furthermore, TDP-43 can either enhance or diminish the targeting of mRNAs by specific miRNAs (Fan et al., 2014; King et al., 2014). These results identify TDP-43 a component of the miRNA pathway and raise the possibility that mutations and/or mislocalization of TDP-43 may disrupt the biogenesis and/or function of miRNAs (**Figure 2**). Indeed, knocking down TDP-43 impairs neuronal differentiation of mouse neuroblastoma cells in culture due in part to a failure to properly process pri-miR-132, a miRNA with extensive links to neuronal differentiation (Kawahara and Mieda-Sato, 2012). Additionally, altered expression profiles of multiple TDP-43-regulated miRNAs is observed in serum and lymphoblast cell lines derived from ALS patients (Freischmidt et al., 2013). Taken together, these studies suggest that miRNA dysfunction downstream of disease-linked TDP-43 alterations could represent an important pathogenic mechanism in neurodegenerative disease and warrants further investigation (**Figure 1**).

Fused in sarcoma is another RNA-binding protein implicated in ALS/FTLD (Kwiatkowski et al., 2009; Vance et al., 2009; Mackenzie et al., 2010). The neuropathology of FUS is remarkably similar to TDP-43 in terms of detection in aberrant cytoplasmic aggregates within affected CNS regions. Unlike TDP-43, FUS shows no direct interaction with Drosha or Dicer (Kawahara and Mieda-Sato, 2012). However, nuclear FUS directly binds pri-mRNA, including the neural outgrowth factor pri-miR-132 (Morlando et al., 2012). Depletion of FUS decreases Drosha levels at miRNA genes, suggesting that FUS facilitates miRNA processing through indirect Drosha interactions (Morlando et al., 2012). FUS also regulates miR-134 and miR-143, both of which display differential neural expression with age (Inukai et al., 2012). Therefore, similarly to TDP-43, FUS may be required in the nucleus to maintain proper miRNA expression in the aging brain (**Figure 2**).

The ATXN2 protein is yet another disease-associated RNA binding protein with a putative role in miRNA function. Aberrant polyglutamine expansion within the human *ATXN2* gene causes spinocerebellar ataxia type 2 (SCA2) and is strongly linked to ALS as well as the atypical parkinsonian disorder called progressive supranuclear palsy (PSP; Pulst et al., 1996; Elden et al., 2010; Ross et al., 2011). Atx2, the *D. melanogaster* ortholog of ATXN2, plays a critical role in long-term habituation of adult olfactory projection neurons via an Ago1- and Me31B- dependent mechanism (McCann et al., 2011). Both proteins are core components of the miRNA pathway in flies, thus identifying Atx2 as an important component of the *D. melanogaster* miRNA pathway (**Figure 2**). Overall, these findings raise the possibility that mutations and/or mislocalization of key RNA binding proteins including TDP-43, FUS, and ATXN2 (**Figure 1**) may lead to the disruption miRNAs that otherwise fulfill critical functions in the brain.

All in all, the miRNA pathway in general and specific miRNAs in particular are emerging as key players in the nervous system as they are linked to neurogenesis, cell survival/longevity, and a healthy/functional lifespan. However, as we will see in the next section, miRNAs are not the only type of ncRNA with such roles.

### Other Small ncRNAs in Neurodegenerative Disease

In addition to miRNAs, a wide variety of small ncRNAs exists. Data linking these latter transcripts with neurodegenerative disorders are relatively limited (Esteller, 2011). However, there is some evidence that neurodegeneration-linked proteins may be responsible for proper regulation of such small ncRNAs. For example, the microprocessor DGCR8 regulates hundreds of small nucleolar RNAs (snoRNAs) independently of Drosha in human cells (Macias et al., 2012). It is important to note that although DGCR8 is also involved in the processing of miRNAs via Drosha interaction, miRNAs are not the most abundant DGCR8 targets (Macias et al., 2012). Furthermore, DGCR8 co-purifies with TDP-43 making it a putative regulator of snoRNA biogenesis (Kawahara and Mieda-Sato, 2012; Macias et al., 2012). Interestingly, defects in snoRNAs are associated with neurodevelopmental disorders such as Prader–Willi syndrome (Sahoo et al., 2008). Furthermore, several snoRNAs are differentially expressed in aged rat brains, so microprocessor action may also be important for the maintenance of proper snoRNA regulation during aging (Wood et al., 2013). Therefore, it is possible that the mislocalization of TDP-43 in ALS, FTLD or AD may impair DGCR8 function. This would in turn inhibit the biogenesis of key neural snoRNAs. Further work is clearly needed to clarify how TDP-43 or other neurodegeneration-related proteins impact snoRNA biogenesis and how this is important for the maintenance of neuronal and brain function.

tRNAs represent another ubiquitous and vital group of small ncRNAs. They recognize RNA codon sequences and allow for the transfer of amino acids, thereby mediating the process of protein synthesis. tRNAs that recognize the same codon, termed isodecoders, are encoded by numerous nuclear genes (Goodenbour and Pan, 2006). These isodecoders are not redundant; mutation of a CNS-specific tRNA isodecoder in mice, *n-Tr20*, is sufficient to cause the loss of a protein, GTPBP2, and results in widespread neurodegeneration (Ishimura et al., 2014). This highlights the importance of individual tRNAs in maintaining neural health. Additionally, mutations in aminoacyl-tRNA synthetases, which match tRNAs with their respective amino acids, leads to neurodegeneration (Lee et al., 2006). Finally, fidelity of the tRNA splicing pathway is important for maintaining proper brain health. Individuals with a mutation in the kinase CLP1 have very early onset neurodegeneration, termed PCH10. This is likely at least in part due to the role CLP1 plays in the maturation of tRNAs as patient neurons are deficient in mature tRNAs and accumulate unspliced pre-tRNAs. Additionally, transfection of tRNA fragments into patient cells exacerbates neurodegenerative phenotypes (Schaffer et al., 2014). All in all, the proper genesis and function of different types of small ncRNAs is important for maintaining neural cell populations and preventing neurodegenerative disease.

### Introduction to lncRNAs

Long non-coding RNAs are a heterogeneously defined group of RNAs, many of which are emerging as regulators of genome expression/stability and modulators of neural function and dysfunction (**Figure 1**; Wu et al., 2013). LncRNAs can be generally defined as non-coding transcripts that are greater than 200 nucleotides in length (Esteller, 2011). Many lncRNAs are molecularly indistinguishable from mRNAs; they are also produced by Pol II and undergo 5′ capping and polyadenylation (Ip and Nakagawa, 2012). Despite the fact that lncRNAs are not as well-characterized as miRNAs or coding transcripts, they actually make up the majority of the mammalian transcriptome (Esteller, 2011). There are a variety of mechanisms through which lncRNAs can impact gene expression. For example, lncRNAs can inhibit or promote transcription through the recruitment of histone modifying complexes or through the binding of specific transcriptional regulators (Geisler and Coller, 2013). lncRNAs are also capable of altering translation through binding to mRNAs and forming duplexes which can promote stability, alter splicing profiles or mask miRNA binding sites (Geisler and Coller, 2013). Here, we highlight how lncRNAs can impact neurodegeneration.

### Antisense lncRNAs Regulate Sense Transcription of Neural Factors

In the context of neurodegenerative diseases, antisense transcripts have emerged as regulators of neural proteins. Antisense transcripts are often lncRNA transcripts that emerge from the opposite strand of a coding RNA region. One such transcript is *BACE1AS*, which is upregulated in AD (Faghihi et al., 2008). *BACE1AS* stabilizes its sense transcript *BACE1* through the formation of an RNA duplex and this increases production of the BACE1 protein (Faghihi et al., 2008). Excess BACE1 then leads to sequential cleavage of the APP to form Aβ peptides, which constitute the amyloid plaques that are characteristic of AD pathobiology (Faghihi et al., 2008). Indeed, both a mouse and a human cell line model of AD demonstrated delayed plaque formation following BACE1AS knockdown (Modarresi et al., 2011; Liu et al., 2014). Therefore, targeting *BACE1AS* may be a promising therapeutic avenue for AD.

A less prevalent neurodegenerative disease, spinocerebellar ataxia type 8 (SCA8), is characterized by a mutation within a DNA region yielding a ncRNA. Here, a lncRNA referred to as *ATXN8OS* has a CTG trinucleotide repeat expansion (Koob et al., 1999; Mutsuddi and Rebay, 2005). This RNA is opposite to the coding strand for a protein referred to as KLHL1 (Nemes et al., 2000). Co-expression analysis of *ATXN8OS* and *KLHL1* RNA in several regions within the human brain suggests that *ATXN8OS* represses the sense transcription of KLHL1 (Chen et al., 2008b). Intriguingly, siRNA-mediated knockdown of KLHL1 in murine pheochromocytoma PC12 cells markedly decreases neurite outgrowth (Seng et al., 2006). Thus, the CTG repeat expansion in *ATXN8OS* may lower KLHL1 expression and this may in turn limit neurite outgrowth and contribute to brain dysfunction.

The neural-specific protein ubiquitin-protein hydrolase UCHL1 is also regulated by its antisense transcript (Carrieri et al., 2012). Individuals carrying UCHL1 mutations, which reduce the hydrolase activity of the enzyme, develop early progressive neurodegeneration (Bilguvar et al., 2013). Interestingly, UCHL1 has also been demonstrated to be oxidatively inactivated in the brains of individuals with PD or AD (Choi et al., 2004; Barrachina et al., 2006). Future work should clarify whether perturbations to the UCHL1 antisense transcript alter UCHL1 expression or function in ways that promote neurodegeneration.

### lncRNAs form Nuclear Bodies that may Protect Against Neurodegeneration

The formation of disease-linked nuclear bodies can be promoted by some lncRNAs, including *NEAT1* (Ip and Nakagawa, 2012). These include the lncRNA *NEAT1*, whose expression is greatly increased in young proliferating cells, HD neurons, and early stage ALS motor neurons (Johnson, 2012; Abdelmohsen et al., 2013; Nishimoto et al., 2013). *NEAT1* is a required component of paraspeckles, which are nuclear ribonucleoprotein-containing bodies (Nakagawa and Hirose, 2012). Interestingly, the formation of paraspeckles has been proposed to represent an important neuronal stress response based on several observations including the fact that *NEAT1* is upregulated in early stage ALS and in the brains of heroin users (Michelhaugh et al., 2011; Nakagawa and Hirose, 2012; Nishimoto et al., 2013). Consistent with a putative role in stress responses, paraspeckles store highly edited RNAs that are rapidly released under various stress conditions (Prasanth et al., 2005). Aberrant stress responses are linked to several neurodegenerative diseases and *NEAT1*/paraspeckle-related defects may thus promote neurodegeneration. Consistent with this notion, FUS localizes to nuclear paraspeckles and is a direct binding partner of *NEAT1* (Nishimoto et al., 2013; Shelkovnikova et al., 2014). In many ALS/FTLD cases, FUS is mutated and mislocalized to the cytoplasm and this may compromise nuclear paraspeckles and their role in responding to stress (Shelkovnikova et al., 2014). Indeed, siRNA-mediated knockdown of FUS eliminates paraspeckle formation in a number of human cell lines (Shelkovnikova et al., 2014). Furthermore, both a transgenic mouse model of ALS-FUS and motor neurons from ALS-FUS patients demonstrated that FUS sequestered vital nuclear paraspeckle proteins into cytoplasmic inclusions (Shelkovnikova et al., 2014). Importantly, cytoplasmic aggregations of FUS have also been reported in a number of trinucleotide repeat disorders including HD (Doi et al., 2008, 2010). Thus, failed nuclear paraspeckle formation may contribute to several neurodegenerative diseases.

Taken together, these studies indicate that functional lncRNAs may naturally counteract neurodegeneration through basic roles in the regulation of gene expression and the modulation of cellular stress responses. In contrast, some lncRNAs as well as other RNAs with either unclear or no natural function may contribute to neurodegeneration as we will discuss in the next section.

## Deleterious Processes Triggered by Non-Coding RNA in Neurodegenerative Disease

### Toxic RNA Sequesters RNA-Binding Proteins

Non-functional RNA can interact with RNA-binding proteins whose functions depend on their specific subcellular distribution. Toxic RNA in patients with neurodegenerative disorders may abnormally sequester these important RNA-binding proteins away from their site of action. For example, the most common genetic cause of both sporadic and familial forms of ALS is a hexanucleotide repeat expansion in an intron of the *C9ORF72* gene (DeJesus-Hernandez et al., 2011; Majounie et al., 2012). This mutation confers a toxic gain-of-function onto the *C9ORF72* transcript: the repeat expansion itself when transplanted to another portion of the genome is sufficient to cause neurodegeneration in *D. melanogaster* (Xu et al., 2013). *C9ORF72* hexanucleotide repeat expansion causes abortive transcripts rich in G-quadruplexes, which are higher order structures built around hydrogen bonded guanine tetrads. Interestingly, FUS associates with G-quadruplexes pointing to a potential connection between FUS and C9ORF72 (Takahama et al., 2013). These G-quadruplexes bind to and sequester the RNA-binding protein nucleolin (Haeusler et al., 2014). With nucleolin distribution altered, cells show distinct signs of nucleolar stress (Haeusler et al., 2014). While it remains unclear if this nucleolar stress directly causes neurodegeneration, these studies demonstrate a novel mechanism through which toxic RNA may lead to neurodegenerative disease via the aberrant sequestration of various RNA-binding proteins.

Many neurodegenerative diseases are characterized by the presence of a trinucleotide repeat expansion. These include HD (which has a CAG expansion in the HTT protein), several types of SCA, and ATXN2-associated ALS (Elden et al., 2010; Nalavade et al., 2013). Trinucleotide repeats can also trigger neurodegeneration via toxic RNA-mediated sequestration of RNA binding proteins. For example, CAG repeat RNA has been shown to bind to and sequester nucleolin (Tsoi et al., 2012; Tsoi and Chan, 2013). While many diseases with CAG repeats have the repeat within coding regions, studies done in the nematode *C. elegans* demonstrated that CAG repeats within a 3′ UTR are sufficient to shorten lifespan and inhibit motility in a repeat-length associated manner (Wang et al., 2011a). Studies using flies also demonstrated that the introduction of an untranslated CAG repeat is sufficient to cause neuronal degeneration (Li et al., 2008). Therefore, toxic RNA is capable of being produced from either coding or non-coding regions and it can act as a toxic agent via sequestration of key RNA binding proteins. This sequestration could lead to nucleolar stress or other ribonucleoprotein-related defects yet to be characterized.

### Repeat-Associated Non-ATG Translation Generates Toxic Dipeptides

In addition to sequestering RNA binding proteins, repeat-containing transcripts can give rise to toxic proteins through translation initiated from within the repeats (**Figure 1**). In *D. melanogaster,* the hexanucleotide repeat present in *C9ORF72* produces repeat rich RNA capable of undergoing RAN translation, in which translation occurs on repeats in all three reading frames even in the absence of a start codon (Mizielinska et al., 2014). This produces toxic dipeptide proteins that lead to cellular degeneration and shortened lifespan in flies (Mizielinska et al., 2014). Several other disease-linked mutations including SCA8-associated *ATXN8* and *ATXN8OS* CAG repeat expansions also generate toxic dipeptides through RAN translation even when full length ATXN8 is not expressed at all (Koob et al., 1999; Zu et al., 2011). Therefore, portions of both the coding sense and non-coding antisense transcript *ATXN8OS* are likely capable of producing toxic dipeptides through RAN translation (Pearson, 2011). Additionally, a CGG repeat in the 5′ UTR of the neurodegenerative disease fragile X-associated tremor/ataxia syndrome (FXTAS)-linked *FMR1* also gives rise to RAN translation products (Todd et al., 2013). Therefore, these observations raise the possibility that a number of diseases associated with GC-rich repeats may be driven by pathogenic processes arising from the accumulation of toxic RAN translation products.

### R-Loops Repress the Expression of Neuroprotective Genes and Threaten Genome Integrity

**FIGURE 3:**
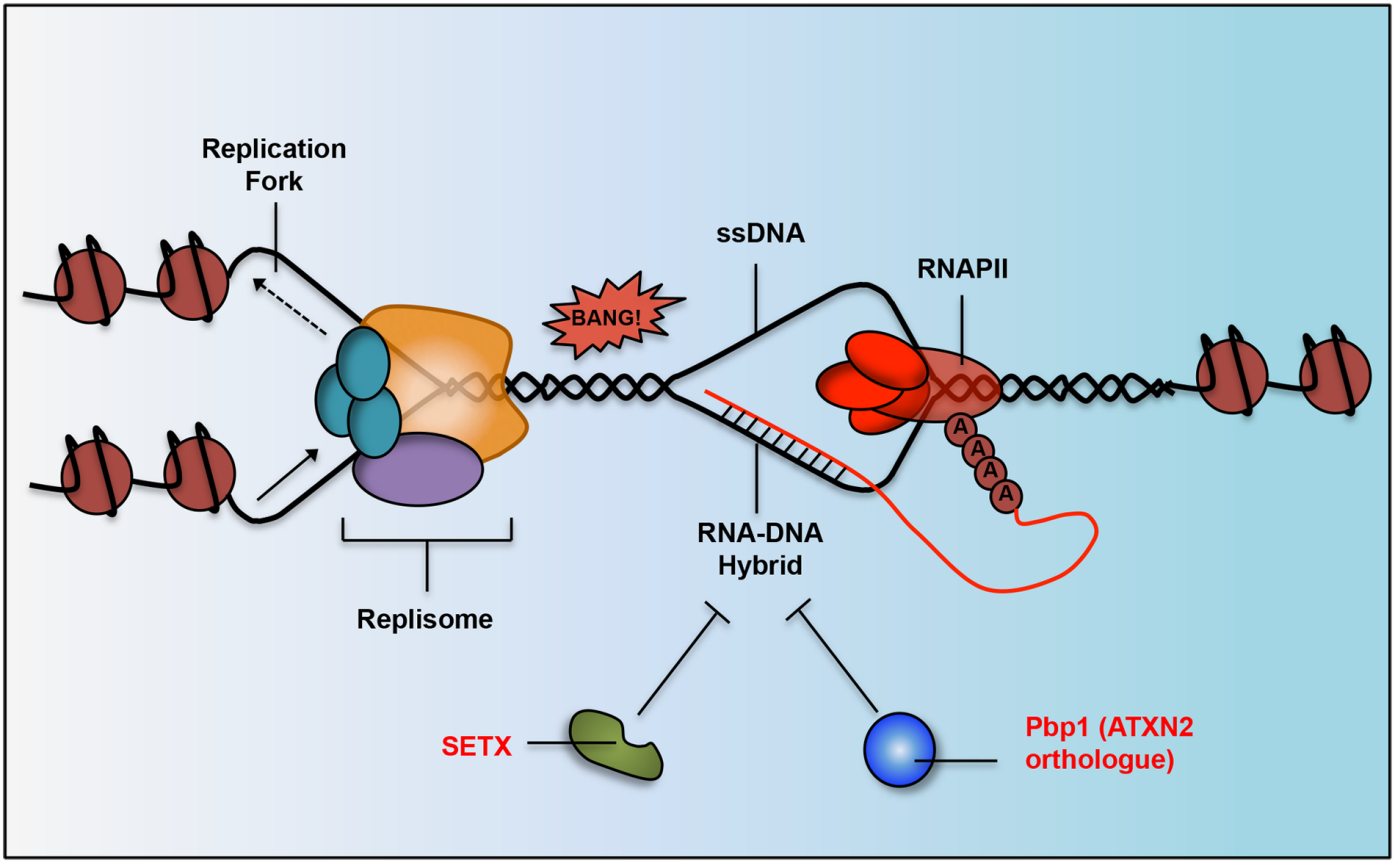
Work done in yeast and humans has determined that neurodegeneration-linked proteins and their orthologs are responsible for RNA–DNA hybrid suppression. R-loop structures form when RNAPII is transcribing DNA and the lagging RNA strand (in red) hybridizes with the DNA strand (in black). SETX (implicated in AOA2 and ALS) has helicase activity and can unwind RNA–DNA hybrid structures. Pbp1 (yeast ortholog of human ATXN2 implicated in SCA2 and ALS) has an RNA-binding domain that inhibits hybrids in some other way, possibly by binding to unwound RNA to prevent it from rehybridizing with its DNA strand. R-loops can lead to double stranded breaks and genomic instability when they collide with advancing replication forks.

Nascent ncRNAs transcripts are capable of hybridizing with their parental DNA strands forming RNA–DNA hybrids (**Figure 3**; Wahba et al., 2011; Aguilera and Garcia-Muse, 2012; Salvi et al., 2014). These RNA–DNA hybrids form structures known as R-loops, which can threaten genome integrity by inducing double stranded breaks and aberrant recombination when collisions occur between R-loops and advancing transcription/replication machineries (Aguilera and Garcia-Muse, 2012). However, R-loops can also regulate the expression of a diverse set of RNAs and are suspected to play a broader role across the genome than it is currently appreciated (Skourti-Stathaki and Proudfoot, 2014).

In several neurodegenerative disorders, R-loops can control the fate of neuroprotective genes (Salvi and Mekhail, 2015). In individuals with fragile X syndrome, a trinucleotide repeat expansion within the promoter of the FXTAS-linked *FMR1* triggers the formation of R-loops, which cause the epigenetic silencing of this gene (Colak et al., 2014). Similarly, R-loops formed upon intronic trinucleotide repeat expansion within the Friedrich’s Ataxia gene *FXN* promote its silencing and are thought to contribute to the disease (Groh et al., 2014). R-loops are also responsible for the abortive transcription observed at the hexanucleotide repeat-expanded *C9ORF72* locus (Haeusler et al., 2014). In addition, data from yeast and *Arabidopsis* point to R-loops as regulators of lncRNA expression but it remains unclear if R-loops regulate neurodegenerative disease-linked lncRNAs such as the AD-linked *BACE1AS* (Nakama et al., 2012; Sun et al., 2013).

Beyond individual genes, some neurodegenerative disorders may encompass broad defects in R-loop or RNA–DNA hybrid regulation. Early onset or juvenile ALS as well as the neurodegenerative disease AOA2 are linked to mutations within SETX, which has emerged as a conserved RNA–DNA helicase from yeast to human (Skourti-Stathaki et al., 2011; Yuce and West, 2013). Loss of SETX function leads to a drastic increase in the number of R-loops formed within cells (Skourti-Stathaki et al., 2011; Yuce and West, 2013). Interestingly, R-loops were easily detected in dividing germ cells but not in post-mitotic brain cells (Yeo et al., 2014). One possibility may be that the level of R-loops driving neural degeneration is too small for detection using present methods. It is also possible that R-loop formation drives disease by disrupting support cells within the nervous system or certain cell types along the neural lineage including neural stem cells. Future work should clarify the intersection between SETX, R-loops and neurodegeneration.

Similar to SETX-ALS/AOA2 links, another possibly early link between R-loops and ATXN2-associated neurodegeneration has recently emerged from a study conducted in the budding yeast *Saccharomyces* *cerevisiae*. It was demonstrated that Pbp1, the yeast ortholog of ATXN2, represses aberrant accumulation of hybrids harboring lncRNA and intergenic DNA within repetitive DNA loci including ribosomal DNA (rDNA) repeats and subtelomeric regions (Salvi et al., 2014). R-loop accumulation in Pbp1-deficient cells triggered aberrant DNA recombination events and shortened replicative lifespan. Whether human ATXN2 functions similarly to suppress deleterious R-loops and if these structures contribute to neuropathology remains to be determined. It is also unclear if the R-loop suppressing functions of ATXN2 and SETX may intersect in ALS and other neurodegenerative diseases.

Therefore, although R-loops can play positive/regulatory roles in the cell, their aberrant regulation may trigger pathological processes leading to neurodegeneration (**Figure 3**). Aberrant R-loop accumulation can lead to the accumulation of toxic abortive transcripts, suppress the expression of neuroprotective factors, or compromise overall genome integrity. The emerging role of R-loops in various diseases may also point to novel avenues for therapeutic intervention.

## Future Questions and Concluding Remarks

While much progress has been made in our understanding of the roles of ncRNAs in neural function, many questions still remain. For example, we know that miRNAs play an important role in neurogenesis, but the role that adult neurogenesis and differentiation plays in many neurodegenerative diseases is unknown. In fact, adult neurogenesis has yet to be detected in most areas of the brain (outside the case of brain damage) including many areas affected in neurodegenerative diseases such as the cerebellum (Ming and Song, 2011) This may be due to detection problems, as the long-lived nature of neurons means that rates of turnover are likely drastically lower when compared to other tissue types. Adult neurogenesis would be an exciting avenue to explore, as it may explain the neural cell-type specificity commonly observed in neurodegenerative diseases. Thus far, it has been demonstrated that premature aging in adult neural stem cells that harbor a LRRK2 mutation characteristic of PD results in a decrease in neurogenesis and improper differentiation (Liu et al., 2012). This hints that Parkinson’s might have defects in adult neurogenesis, although it is also possible that defects in differentiation that occur early in life due to this mutation might predispose neurons to premature death much later in life. Future work examining if neural stem cells are prematurely dying or improperly differentiating in neurodegenerative settings are needed.

More broadly, the reason that neurodegenerative diseases do not manifest until late in life is poorly understood. Why is HD onset typically in the fourth decade when the related HTT mutation is present from birth? One model is that most heritable polymorphisms in neurodegenerative disease-linked factors cause minor molecular defects, as gross genetic failures would exit the gene pool by natural selection. Quality control of protein synthesis decreases with time and there is therefore an increased occurrence/accumulation of misfolded proteins with age (Gaczynska et al., 2001). Such misfolding can contribute to neurodegeneration. Consistently, the unfolded protein response (UPR), an apoptotic defense mechanism against the accumulation of misfolded proteins within the cell, is hyperactivated, perhaps aberrantly, in a number of neurodegenerative diseases including HD, AD, and PD (Halliday and Mallucci, 2014). Another non-exclusive possibility is that several nuclear RNA binding proteins associated with neurodegeneration such as TDP-43 and ATXN2 are recruited to cytoplasmic stress granules and this may prevent their proper RNA regulatory function (Colombrita et al., 2009; Damrath et al., 2012). Stress granule formation under oxidative stress and clearance by autophagy is respectively increased and decreased with age (Colombrita et al., 2009; Buchan et al., 2013; Szafranski and Mekhail, 2014). Thus, the aberrant sequestration of proteins such as TDP-43 and ATXN2 may increase with age and small defects may become amplified over time leading to neurodegeneration. Oxidative stress is also known to cause senescence within astroctyes (Bitto et al., 2011). Senescent astrocytes are enriched in patients with AD relative to age-matched controls (Bhat et al., 2012). This may contribute to disease as these astrocytes have a senescence-associated secretory phenotype wherein they release inflammatory cytokines such as IL-6 (Bhat et al., 2012). Given that astrocytes are the most abundant cell-type in the brain and are more susceptible to oxidative stress than other cell types such as fibroblasts, it is likely that astrocyte senescence is another age-related risk factor for neurodegenerative diseases (Bitto et al., 2011). Future work will undoubtedly clarify the link between aging and neurodegeneration.

Many studies also hinted at links that need further clarification. For example, is miR-34a repressing Bcl2 and SIRT1 *in vivo* and is this repression leading to neurodegeneration? Is ATXN8OS regulating the transcription of KLHL1? Studies examining orthologs of human transcripts or proteins should also be repeated in human models. For example, miR-8 was found to regulate atrophin levels to prevent neurodegeneration in flies (Karres et al., 2007). Both miR-8 and atrophin are conserved; so is the link to neurodegeneration also conserved? Studies reporting that ATXN2 orthologs impact *D. melanogaster* RISC and *S. cerevisiae* R-loop accumulation should serve as foundation for similar studies conducted in mammalian/human model systems. The link between paraspeckles and neuronal stress response also needs to be clarified, as the current literature is mostly correlative.

Overall, commendable gains have been made in understanding the pathobiological processes underlying various neurodegenerative diseases. However, significant research remains to be done in various organisms in order to fully decipher human disease mechanisms. Such mechanistic studies promise to uncover new avenues for targeted therapeutics. For example, understanding the role of neural stem cells in neurodegenerative disease could allow for the development and perfection of stem cell based therapies. Currently, neural stem cell injection therapy for ALS has successfully demonstrated safety and is moving to Phase II trials (Feldman et al., 2014). However, we do not yet know if these stem cells would be resistant to the factors that induce cell death in motor neurons or whether stem cell defects are present in ALS neurons. Other possible therapeutic avenues include ASOs against proteins that are misfolded in neurodegenerative disease. Indeed, studies targeting HTT with ASOs have demonstrated efficacy in primate models (Kordasiewicz et al., 2012). ASO-mediated targeting of *C9ORF72* suppressed ALS-linked defects typically observed in patient-derived induced pluripotent stem cells (Donnelly et al., 2013). On another front, it is also possible to target specific miRNAs. For example, repressing miR-34 may alleviate some of the brain degeneration seen in AD (**Table 1**; Wang et al., 2009; Li et al., 2011b). While no drugs targeting miRNAs in neurodegenerative disease have been developed yet, trials have demonstrated the safety and efficacy of targeted inhibition of a specific miRNA in the treatment of Hepatitis C (Janssen et al., 2013).

As illustrated in this article, ncRNAs, neurodegenerative diseases, and aging are interconnected. miRNAs, lncRNAs, and related factors can impact the nervous system in various positive and negative manners depending on the precise underlying molecular processes. Continued efforts to understand the molecular pathways underlying natural neural function, devastating neurodegenerative diseases and their connection to aging will undoubtedly lead to the development of smarter therapeutics.

## ABBREVIATIONS

AD: Alzheimer’s disease
ALS: amyotrophic lateral sclerosis
AOA2: ataxia with oculomotor apraxia type 2
ASO: antisense oligonucleotide
ATXN: Ataxin
BACE-1: beta-secretase 1
BCL: B-cell lymphoma
C9ORF72: Chromosome 9 open reading frame 72
CLP1: cleavage and polyadenylation factor 1 subunit 1
CNS: central nervous system
DN: dopaminergic neuron
FTLD: frontotemporal lobar degeneration
FUS: fused in sarcoma
HD: Huntington’s disease
KLHL1: Kelch-like 1
lncRNA: long non-coding RNA
miRNA: microRNA
ncRNA: non-coding RNA
NEAT1: nuclear enriched abundant transcript 1
PCH: pontocerebellar hypoplasia
PD: Parkinson’s disease
RAN: repeat-associated non-ATG
RISC: RNA-induced silencing complex
SCA: spinocerebellar ataxia
SETX: Senataxin
SIRT1: Sirtuin 1
TDP-43: TAR-DNA binding protein 43

## References

[B1] Abdelmohsen K. Panda A. Kang M. J. Xu J. Selimyan R. Yoon J. H. (2013). Senescence-associated lncRNAs: senescence-associated long noncoding RNAs. *Aging Cell* 12 890–900 10.1111/acel.12115 23758631 PMC3773026

[B2] Abe M. Bonini N. M. (2013). MicroRNAs and neurodegeneration: role and impact. *Trends Cell Biol.* 23 30–36 10.1016/j.tcb.2012.08.013 23026030 PMC3540990

[B3] Aguilera A. Garcia-Muse T. (2012). R loops: from transcription byproducts to threats to genome stability. *Mol. Cell* 46 115–124 10.1016/j.molcel.2012.04.009 22541554

[B4] Araki T. Sasaki Y. Milbrandt J. (2004). Increased nuclear NAD biosynthesis and SIRT1 activation prevent axonal degeneration. *Science* 305 1010–1013 10.1126/science.1098014 15310905

[B5] Bak M. Silahtaroglu A. Moller M. Christensen M. Rath M. F. Skryabin B. (2008). MicroRNA expression in the adult mouse central nervous system. *RNA* 14 432–444 10.1261/rna.783108 18230762 PMC2248253

[B6] Baloh R. H. (2012). How do the RNA-binding proteins TDP-43 and FUS relate to amyotrophic lateral sclerosis and frontotemporal degeneration, and to each other? *Curr. Opin. Neurol.* 25 701–707 10.1097/Wco.0b013e32835a269b 23041957

[B7] Barrachina M. Castano E. Dalfo E. Maes T. Buesa C. Ferrer I. (2006). Reduced ubiquitin C-terminal hydrolase-1 expression levels in dementia with Lewy bodies. *Neurobiol. Dis.* 22 265–273 10.1016/j.nbd.2005.11.005 16380264

[B8] Bartel D. P. (2004). MicroRNAs: genomics, biogenesis, mechanism, and function. *Cell* 116 281–297 10.1016/S0092-8674(04)00045-5 14744438

[B9] Bhat R. Crowe E. P. Bitto A. Moh M. Katsetos C. D. Garcia F. U. (2012). Astrocyte senescence as a component of Alzheimer’s disease. *PLoS ONE* 7:e45069 10.1371/journal.pone.0045069 PMC3440417 22984612

[B10] Bilen J. Liu N. Burnett B. G. Pittman R. N. Bonini N. M. (2006). MicroRNA pathways modulate polyglutamine-induced neurodegeneration. *Mol. Cell* 24 157–163 10.1016/j.molcel.2006.07.030 17018300

[B11] Bilguvar K. Tyagi N. K. Ozkara C. Tuysuz B. Bakircioglu M. Choi M. (2013). Recessive loss of function of the neuronal ubiquitin hydrolase UCHL1 leads to early-onset progressive neurodegeneration. *Proc. Natl. Acad. Sci. U.S.A.* 110 3489–3494 10.1073/pnas.1222732110 23359680 PMC3587195

[B12] Bitto A. Sell C. Crowe E. Lorenzini A. Malaguti M. Torres C. (2011). Stress-induced senescence in human and rodent astrocytes. *Exp. Gerontol.* 46 213–213 10.1016/j.exger.2010.11.028 20620137

[B13] Bremer J. O’Connor T. Tiberi C. Rehrauer H. Weis J. Aguzzi A. (2010). Ablation of dicer from Murine Schwann Cells increases their proliferation while blocking myelination. *PLoS ONE* 5:e12450 10.1371/journal.pone.0012450 PMC2929198 20805985

[B14] Buchan J. R. Kolaitis R. M. Taylor J. P. Parker R. (2013). Eukaryotic stress granules are cleared by autophagy and Cdc48/VCP function. *Cell* 153 1461–1474 10.1016/j.cell.2013.05.037 23791177 PMC3760148

[B15] Carrieri C. Cimatti L. Biagioli M. Beugnet A. Zucchelli S. Fedele S. (2012). Long non-coding antisense RNA controls Uchl1 translation through an embedded SINEB2 repeat. *Nature* 491 454–457 10.1038/Nature11508 23064229

[B16] Cheloufi S. Dos Santos C. O. Chong M. M. W. Hannon G. J. (2010). A Dicer-independent miRNA biogenesis pathway that requires Ago catalysis. *Nature* 465 U584–U576 10.1038/Nature09092 PMC2995450 20424607

[B17] Chen D. Steele A. D. Hutter G. Bruno J. Govindarajan A. Easlon E. (2008a). The role of calorie restriction and SIRT1 in prion-mediated neurodegeneration. *Exp. Gerontol.* 43 1086–1093 10.1016/j.exger.2008.08.050 18799131 PMC2735260

[B18] Chen W. L. Lin J. W. Huang H. J. Wang S. M. Su M. T. Lee-Chen G. J. (2008b). SCA8 mRNA expression suggests an antisense regulation of KLHL1 and correlates to SCA8 pathology. *Brain Res.* 1233 176–184 10.1016/j.brainres.2008.07.096 18708037

[B19] Chen J. A. Wichterle H. (2012). Apoptosis of limb innervating motor neurons and erosion of motor pool identity upon lineage specific dicer inactivation. *Front. Neurosci.* 6:69 10.3389/fnins.2012.00069 PMC3354549 22629237

[B20] Choi J. Levey A. I. Weintraub S. T. Rees H. D. Gearing M. Chin L. S. (2004). Oxidative modifications and down-regulation of ubiquitin carboxyl-terminal hydrolase L1 associated with idiopathic Parkinson’s and Alzheimer’s diseases. *J. Biol. Chem.* 279 13256–13264 10.1074/jbc.M314124200 14722078

[B21] Choi P. S. Zakhary L. Choi W. Y. Caron S. Alvarez-Saavedra E. Miska E. A. (2008). Members of the miRNA-200 family regulate olfactory neurogenesis. *Neuron* 57 41–55 10.1016/j.neuron.2007.11.018 18184563 PMC2204047

[B22] Colak D. Zaninovic N. Cohen M. S. Rosenwaks Z. Yang W. Y. Gerhardt J. (2014). Promoter-bound trinucleotide repeat mRNA drives epigenetic silencing in fragile X syndrome. *Science* 343 1002–1005 10.1126/science.1245831 24578575 PMC4357282

[B23] Colombrita C. Zennaro E. Fallini C. Weber M. Sommacal A. Buratti E. (2009). TDP-43 is recruited to stress granules in conditions of oxidative insult. *J. Neurochem.* 111 1051–1061 10.1111/j.1471-4159.2009.06383.x 19765185

[B24] Damiani D. Alexander J. J. O’Rourke J. R. Mcmanus M. Jadhav A. P. Cepko C. L. (2008). Dicer inactivation leads to progressive functional and structural degeneration of the mouse retina. *J. Neurosci.* 28 4878–4887 10.1523/JNEUROSCI.0828-08.2008 18463241 PMC3325486

[B25] Damrath E. Heck M. V. Gispert S. Azizov M. Nowock J. Seifried C. (2012). ATXN2-CAG42 sequesters PABPC1 into insolubility and induces FBXW8 in cerebellum of old Ataxic Knock-in mice. *PLoS Genet.* 8:e1002920 10.1371/journal.pgen.1002920 PMC3431311 22956915

[B26] Davis T. H. Cuellar T. L. Koch S. M. Barker A. J. Harfe B. D. Mcmanus M. T. (2008). Conditional loss of dicer disrupts cellular and tissue morphogenesis in the cortex and hippocampus. *J. Neurosci.* 28 4322–4330 10.1523/JNEUROSCI.4815-07.2008 18434510 PMC3844796

[B27] DeJesus-Hernandez M. Mackenzie I. R. Boeve B. F. Boxer A. L. Baker M. Rutherford N. J. (2011). Expanded GGGGCC hexanucleotide repeat in noncoding region of C9ORF72 causes chromosome 9p-linked FTD and ALS. *Neuron* 72 245–256 10.1016/j.neuron.2011.09.011 21944778 PMC3202986

[B28] Di Sante G. Wang L. Wang C. Jiao X. Casimiro M. C. Chen K. (2015). Sirt1-deficient mice have hypogonadotropic hypogonadism due to defective GnRH neuronal migration. *Mol. Endocrinol.* 29 200–212 10.1210/me.2014-1228 25545407 PMC4318884

[B29] Djebali S. Davis C. A. Merkel A. Dobin A. Lassmann T. Mortazavi A. (2012). Landscape of transcription in human cells. *Nature* 489 101–108 10.1038/nature11233 22955620 PMC3684276

[B30] Doi H. Koyano S. Suzuki Y. Nukina N. Kuroiwa Y. (2010). The RNA-binding protein FUS/TLS is a common aggregate-interacting protein in polyglutamine diseases. *Neurosci. Res.* 66 131–133 10.1016/j.neures.2009.10.004 19833157

[B31] Doi H. Okamura K. Bauer P. O. Furukawa Y. Shimizu H. Kurosawa M. (2008). RNA-binding protein TLS is a major nuclear aggregate-interacting protein in huntingtin exon 1 with expanded polyglutamine-expressing cells. *J. Biol. Chem.* 283 6489–6500 10.1074/jbc.M705306200 18167354

[B32] Donnelly C. J. Zhang P. W. Pham J. T. Haeusler A. R. Mistry N. A. Vidensky S. (2013). RNA toxicity from the ALS/FTD C9ORF72 expansion is mitigated by antisense intervention. *Neuron* 80 415–428 10.1016/j.neuron.2013.10.015 24139042 PMC4098943

[B33] Eacker S. M. Dawson T. M. Dawson V. L. (2009). Understanding microRNAs in neurodegeneration. *Nat. Rev. Neurosci.* 10 837–841 10.1038/nrn2726 19904280 PMC4120241

[B34] Elden A. C. Kim H. J. Hart M. P. Chen-Plotkin A. S. Johnson B. S. Fang X. (2010). Ataxin-2 intermediate-length polyglutamine expansions are associated with increased risk for ALS. *Nature* 466 1069–1075 10.1038/nature09320 20740007 PMC2965417

[B35] Esteller M. (2011). Non-coding RNAs in human disease. *Nat. Rev. Genet.* 12 861–874 10.1038/Nrg3074 22094949

[B36] Faghihi M. A. Modarresi F. Khalil A. M. Wood D. E. Sahagan B. G. Morgan T. E. (2008). Expression of a noncoding RNA is elevated in Alzheimer’s disease and drives rapid feed-forward regulation of beta-secretase. *Nat. Med.* 14 723–730 10.1038/Nm1784 18587408 PMC2826895

[B37] Fan Z. Chen X. Chen R. (2014). Transcriptome-wide analysis of TDP-43 binding small RNAs identifies miR-NID1 (miR-8485), a novel miRNA that represses *NRXN1* expression. *Genomics* 103 76–82 10.1016/j.ygeno.2013.06.006 23827811

[B38] Feldman E. L. Boulis N. M. Hur J. Johe K. Rutkove S. B. Federici T. (2014). Intraspinal neural stem cell transplantation in amyotrophic lateral sclerosis: phase 1 trial outcomes. *Ann. Neurol.* 75 363–373 10.1002/ana.24113 24510776 PMC4005820

[B39] Freischmidt A. Muller K. Ludolph A. C. Weishaupt J. H. (2013). Systemic dysregulation of TDP-43 binding microRNAs in amyotrophic lateral sclerosis. *Acta Neuropathol. Commun.* 1:42 10.1186/2051-5960-1-42 PMC3893596 24252274

[B40] Gaczynska M. Osmulski P. A. Ward W. F. (2001). Caretaker or undertaker? The role of the proteasome in aging. *Mech. Ageing Dev.* 122 235–254 10.1016/S0047-6374(00)00246-3 11311314

[B41] Geisler S. Coller J. (2013). RNA in unexpected places: long non-coding RNA functions in diverse cellular contexts. *Nat. Rev. Mol. Cell Biol.* 14 699–712 10.1038/nrm3679 24105322 PMC4852478

[B42] Giraldez A. J. Cinalli R. M. Glasner M. E. Enright A. J. Thomson J. M. Baskerville S. (2005). MicroRNAs regulate brain morphogenesis in zebrafish. *Science* 308 833–838 10.1126/science.1109020 15774722

[B43] Goodenbour J. M. Pan T. (2006). Diversity of tRNA genes in eukaryotes. *Nucleic Acids Res.* 34 6137–6146 10.1093/Nar/Gkl725 17088292 PMC1693877

[B44] Graff J. Kahn M. Samiei A. Gao J. Ota K. T. Rei D. (2013). A Dietary regimen of Caloric Restriction or pharmacological activation of SIRT1 to delay the onset of neurodegeneration. *J. Neurosci.* 33 8951–8960 10.1523/Jneurosci.5657-12.2013 23699506 PMC3775567

[B45] Groh M. Lufino M. M. Wade-Martins R. Gromak N. (2014). R-loops associated with triplet repeat expansions promote gene silencing in Friedreich ataxia and fragile X syndrome. *PLoS Genet* 10:e1004318 10.1371/journal.pgen.1004318 PMC4006715 24787137

[B46] Ha M. Kim V. N. (2014). Regulation of microRNA biogenesis. *Nat. Rev. Mol. Cell Biol.* 15 509–524 10.1038/Nrm3838 25027649

[B47] Haeusler A. R. Donnelly C. J. Periz G. Simko E. A. J. Shaw P. G. Kim M. S. (2014). C9orf72 nucleotide repeat structures initiate molecular cascades of disease. *Nature* 507 195–200 10.1038/Nature13124 24598541 PMC4046618

[B48] Halliday M. Mallucci G. R. (2014). Targeting the unfolded protein response in neurodegeneration: a new approach to therapy. *Neuropharmacology* 76(Pt A) 169–174 10.1016/j.neuropharm.2013.08.034 24035917

[B49] Haramati S. Chapnik E. Sztainberg Y. Eilam R. Zwang R. Gershoni N. (2010). miRNA malfunction causes spinal motor neuron disease. *Proc. Natl. Acad. Sci. U.S.A.* 107 13111–13116 10.1073/pnas.1006151107 20616011 PMC2919953

[B50] Hardiman O. van den Berg L. H. Kiernan M. C. (2011). Clinical diagnosis and management of amyotrophic lateral sclerosis. *Nat. Rev. Neurol.* 7 639–649 10.1038/nrneurol.2011.153 21989247

[B51] He L. Hannon G. J. (2004). MicroRNAs: small RNAs with a big role in gene regulation. *Nat. Rev. Genet.* 5 522–531 10.1038/nrg1379 15211354

[B52] Hebert S. S. Horre K. Nicolai L. Papadopoulou A. S. Mandemakers W. Silahtaroglu A. N. (2008). Loss of microRNA cluster miR-29a/b-1 in sporadic Alzheimer’s disease correlates with increased BACE1/beta-secretase expression. *Proc. Natl. Acad. Sci. U.S.A.* 105 6415–6420 10.1073/pnas.0710263105 18434550 PMC2359789

[B53] Huang P. S. Son J. H. Abbott L. C. Winzer-Serhan U. H. (2011). Regulated expression of neuronal sirt1 and related genes by aging and neuronal beta 2-containing nicotinic cholinergic receptors. *Neuroscience* 196 189–202 10.1016/j.neuroscience.2011.09.007 21939740

[B54] Huang T. Liu Y. Huang M. Zhao X. Cheng L. (2010). Wnt1-cre-mediated conditional loss of dicer results in malformation of the midbrain and cerebellum and failure of neural crest and dopaminergic differentiation in mice. *J. Mol. Cell Biol.* 2 152–163 10.1093/jmcb/mjq008 20457670

[B55] Inukai S. De Lencastre A. Turner M. Slack F. (2012). Novel microRNAs differentially expressed during aging in the mouse brain. *PLoS ONE* 7:e40028 10.1371/journal.pone.0040028 PMC3402511 22844398

[B56] Ip J. Y. Nakagawa S. (2012). Long non-coding RNAs in nuclear bodies. *Dev. Growth Differ.* 54 44–54 10.1111/j.1440-169X.2011.01303.x 22070123

[B57] Ishimura R. Nagy G. Dotu I. Zhou H. H. Yang X. L. Schimmel P. (2014). Ribosome stalling induced by mutation of a CNS-specific tRNA causes neurodegeneration. *Science* 345 455–459 10.1126/science.1249749 25061210 PMC4281038

[B58] Janssen H. L. Reesink H. W. Lawitz E. J. Zeuzem S. Rodriguez-Torres M. Patel K. (2013). Treatment of HCV infection by targeting microRNA. *N. Engl. J. Med.* 368 1685–1694 10.1056/NEJMoa1209026 23534542

[B59] Jiang M. L. Wang J. W. Fu J. R. Du L. Jeong H. West T. (2012). Neuroprotective role of Sirt1 in mammalian models of Huntington’s disease through activation of multiple Sirt1 targets. *Nat. Med.* 18 153–158 10.1038/Nm.2558 22179319 PMC4551453

[B60] Johnson R. (2012). Long non-coding RNAs in Huntington’s disease neurodegeneration. *Neurobiol. Dis.* 46 245–254 10.1016/j.nbd.2011.12.006 22202438

[B61] Jovicic A. Roshan R. Moisoi N. Pradervand S. Moser R. Pillai B. (2013). Comprehensive expression analyses of neural cell-type-specific miRNAs identify new determinants of the specification and maintenance of neuronal phenotypes. *J. Neurosci.* 33 5127–5137 10.1523/JNEUROSCI.0600-12.2013 23516279 PMC6705001

[B62] Kabashi E. Valdmanis P. N. Dion P. Spiegelman D. Mcconkey B. J. Vande Velde C. (2008). TARDBP mutations in individuals with sporadic and familial amyotrophic lateral sclerosis. *Nat. Genet.* 40 572–574 10.1038/ng.132 18372902

[B63] Karres J. S. Hilgers V. Carrera I. Treisman J. Cohen S. M. (2007). The conserved microRNA miR-8 tunes atrophin levels to prevent neurodegeneration in *Drosophila*. *Cell* 131 136–145 10.1016/j.cell.2007.09.020 17923093

[B64] Kawahara Y. Mieda-Sato A. (2012). TDP-43 promotes microRNA biogenesis as a component of the Drosha and Dicer complexes. *Proc. Natl. Acad. Sci. U.S.A.* 109 3347–3352 10.1073/pnas.1112427109 22323604 PMC3295278

[B65] Khanna S. Rink C. Ghoorkhanian R. Gnyawali S. Heigel M. Wijesinghe D. S. (2013). Loss of miR-29b following acute ischemic stroke contributes to neural cell death and infarct size. *J. Cereb. Blood Flow Metab.* 33 1197–1206 10.1038/jcbfm.2013.68 23632968 PMC3734770

[B66] Kim D. Nguyen M. D. Dobbin M. M. Fischer A. Sananbenesi F. Rodgers J. T. (2007a). SIRT1 deacetylase protects against neurodegeneration in models for Alzheimer’s disease and amyotrophic lateral sclerosis. *EMBO J.* 26 3169–3179 10.1038/sj.emboj.7601758 17581637 PMC1914106

[B67] Kim J. Inoue K. Ishii J. Vanti W. B. Voronov S. V. Murchison E. (2007b). A MicroRNA feedback circuit in midbrain dopamine neurons. *Science* 317 1220–1224 10.1126/science.1140481 17761882 PMC2782470

[B68] King I. N. Yartseva V. Salas D. Kumar A. Heidersbach A. Ando D. M. (2014). The RNA-binding protein TDP-43 selectively disrupts microRNA-1/206 incorporation into the RNA-induced silencing complex. *J. Biol. Chem.* 289 14263–14271 10.1074/jbc.M114.561902 24719334 PMC4022891

[B69] Kole A. J. Swahari V. Hammond S. M. Deshmukh M. (2011). miR-29b is activated during neuronal maturation and targets BH3-only genes to restrict apoptosis. *Genes Dev.* 25 125–130 10.1101/gad.1975411 21245165 PMC3022258

[B70] Koob M. D. Moseley M. L. Schut L. J. Benzow K. A. Bird T. D. Day J. W. (1999). An untranslated CTG expansion causes a novel form of spinocerebellar ataxia (SCA8). *Nat. Genet.* 21 379–384 10.1038/7710 10192387

[B71] Kordasiewicz H. B. Stanek L. M. Wancewicz E. V. Mazur C. Mcalonis M. M. Pytel K. A. (2012). Sustained therapeutic reversal of Huntington’s disease by transient repression of huntingtin synthesis. *Neuron* 74 1031–1044 10.1016/j.neuron.2012.05.009 22726834 PMC3383626

[B72] Kwiatkowski T. J. Jr. Bosco D. A. Leclerc A. L. Tamrazian E. Vanderburg C. R. Russ C. (2009). Mutations in the FUS/TLS gene on chromosome 16 cause familial amyotrophic lateral sclerosis. *Science* 323 1205–1208 10.1126/science.1166066 19251627

[B73] Lagier-Tourenne C. Cleveland D. W. (2009). Rethinking ALS: the FUS about TDP-43. *Cell* 136 1001–1004 10.1016/j.cell.2009.03.006 19303844 PMC3110083

[B74] Lee J. W. Beebe K. Nangle L. A. Jang J. S. Longo-Guess C. M. Cook S. A. (2006). Editing-defective tRNA synthetase causes protein misfolding and neurodegeneration. *Nature* 443 50–55 10.1038/Nature05096 16906134

[B75] Lee S. T. Chu K. Im W. S. Yoon H. J. Im J. Y. Park J. E. (2011). Altered microRNA regulation in Huntington’s disease models. *Exp. Neurol.* 227 172–179 10.1016/j.expneurol.2010.10.012 21035445

[B76] Lee Y. Jeon K. Lee J. T. Kim S. Kim V. N. (2002). MicroRNA maturation: stepwise processing and subcellular localization. *EMBO J.* 21 4663–4670 10.1093/emboj/cdf476 12198168 PMC126204

[B77] Li L. B. Yu Z. M. Teng X. Y. Bonini N. M. (2008). RNA toxicity is a component of ataxin-3 degeneration in *Drosophila*. *Nature* 453 U1107–U1109 10.1038/Nature06909 PMC2574630 18449188

[B78] Li N. Bates D. J. An J. Terry D. A. Wang E. (2011a). Up-regulation of key microRNAs, and inverse down-regulation of their predicted oxidative phosphorylation target genes, during aging in mouse brain. *Neurobiol. Aging* 32 944–955 10.1016/j.neurobiolaging.2009.04.020 19487051

[B79] Li X. Khanna A. Li N. Wang E. (2011b). Circulatory miR34a as an RNAbased, noninvasive biomarker for brain aging. *Aging (Albany, NY)* 3 985–1002. 22064828 10.18632/aging.100371 PMC3229974

[B80] Liu G. H. Qu J. Suzuki K. Nivet E. Li M. Montserrat N. (2012). Progressive degeneration of human neural stem cells caused by pathogenic LRRK2. *Nature* 491 603–607 10.1038/nature11557 23075850 PMC3504651

[B81] Liu T. Huang Y. Chen J. Chi H. Yu Z. Wang J. (2014). Attenuated ability of BACE1 to cleave the amyloid precursor protein via silencing long noncoding RNA BACE1AS expression. *Mol. Med. Rep.* 10 1275–1281 10.3892/mmr.2014.2351 24970022 PMC4121421

[B82] Macias S. Plass M. Stajuda A. Michlewski G. Eyras E. Caceres J. F. (2012). DGCR8 HITS-CLIP reveals novel functions for the Microprocessor. *Nat. Struct. Mol. Biol.* 19 760–766 10.1038/Nsmb.2344 22796965 PMC3442229

[B83] Mackenzie I. R. A. Rademakers R. Neumann M. (2010). TDP-43 and FUS in amyotrophic lateral sclerosis and frontotemporal dementia. *Lancet Neurol.* 9 995–1007 10.1016/S1474-4422(10)70195-2 20864052

[B84] Majounie E. Renton A. E. Mok K. Dopper E. G. Waite A. Rollinson S. (2012). Frequency of the C9orf72 hexanucleotide repeat expansion in patients with amyotrophic lateral sclerosis and frontotemporal dementia: a cross-sectional study. *Lancet Neurol.* 11 323–330 10.1016/S1474-4422(12)70043-1 22406228 PMC3322422

[B85] Mattson M. P. (2004). Pathways towards and away from Alzheimer’s disease. *Nature* 430 631–639 10.1038/Nature02621 15295589 PMC3091392

[B86] McCann C. Holohan E. E. Das S. Dervan A. Larkin A. Lee J. A. (2011). The Ataxin-2 protein is required for microRNA function and synapse-specific long-term olfactory habituation. *Proc. Natl. Acad. Sci. U.S.A.* 108 E655–E662 10.1073/pnas.1107198108 21795609 PMC3169144

[B87] Michelhaugh S. K. Lipovich L. Blythe J. Jia H. Kapatos G. Bannon M. J. (2011). Mining Affymetrix microarray data for long non-coding RNAs: altered expression in the nucleus accumbens of heroin abusers. *J. Neurochem.* 116 459–466 10.1111/j.1471-4159.2010.07126.x 21128942 PMC3061462

[B88] Ming G. L. Song H. (2011). Adult neurogenesis in the mammalian brain: significant answers and significant questions. *Neuron* 70 687–702 10.1016/j.neuron.2011.05.001 21609825 PMC3106107

[B89] Mizielinska S. Gronke S. Niccoli T. Ridler C. E. Clayton E. L. Devoy A. (2014). C9orf72 repeat expansions cause neurodegeneration in *Drosophila* through arginine-rich proteins. *Science* 345 1192–1194 10.1126/science.1256800 25103406 PMC4944841

[B90] Modarresi F. Faghihi M. A. Patel N. S. Sahagan B. G. Wahlestedt C. Lopez-Toledano M. A. (2011). Knockdown of BACE1-AS nonprotein-coding transcript modulates beta-amyloid-related hippocampal neurogenesis. *Int. J. Alzheimers Dis.* 2011 929042 10.4061/2011/929042 PMC3139208 21785702

[B91] Morlando M. Modigliani S. D. Torrelli G. Rosa A. Di Carlo V. Caffarelli E. (2012). FUS stimulates microRNA biogenesis by facilitating co-transcriptional Drosha recruitment. *EMBO J.* 31 4502–4510 10.1038/emboj.2012.319 23232809 PMC3545295

[B92] Mutsuddi M. Rebay I. (2005). Molecular genetics of spinocerebellar ataxia type 8 (SCA8). *RNA Biol.* 2 49–52 10.4161/rna.2.2.1682 17132942

[B93] Nakagawa S. Hirose T. (2012). Paraspeckle nuclear bodies-useful uselessness? *Cell. Mol. Life Sci.* 69 3027–3036 10.1007/s00018-012-0973-x 22476590 PMC3428521

[B94] Nakama M. Kawakami K. Kajitani T. Urano T. Murakami Y. (2012). DNA-RNA hybrid formation mediates RNAi-directed heterochromatin formation. *Genes Cells* 17 218–233 10.1111/j.1365-2443.2012.01583.x 22280061

[B95] Nalavade R. Griesche N. Ryan D. P. Hildebrand S. Krauss S. (2013). Mechanisms of RNA-induced toxicity in CAG repeat disorders. *Cell Death Dis.* 4:e752 10.1038/cddis.2013.276 PMC3763438 23907466

[B96] Nemes J. P. Benzow K. A. Moseley M. L. Ranum L. P. W. Koob M. D. (2000). The SCA8 transcript is an antisense RNA to a brain-specific transcript encoding a novel actin-binding protein (KLHL1) (vol 9 pg 1543 2000). *Hum. Mol. Genet.* 9 2777–2777 10.1093/hmg/9.10.1543 10888605

[B97] Neumann M. Sampathu D. M. Kwong L. K. Truax A. C. Micsenyi M. C. Chou T. T. (2006). Ubiquitinated TDP-43 in frontotemporal lobar degeneration and amyotrophic lateral sclerosis. *Science* 314 130–133 10.1126/science.1134108 17023659

[B98] Nishimoto Y. Nakagawa S. Hirose T. Okano H. J. Takao M. Shibata S. (2013). The long non-coding RNA nuclear-enriched abundant transcript 1_2 induces paraspeckle formation in the motor neuron during the early phase of amyotrophic lateral sclerosis. *Mol. Brain* 6:31 10.1186/1756-6606-6-31 PMC3729541 23835137

[B99] O’Brien R. J. Wong P. C. (2011). Amyloid precursor protein processing and Alzheimer’s disease. *Annu. Rev. Neurosci.* 34 185–204 10.1146/annurev-neuro-061010-113613 21456963 PMC3174086

[B100] Palazzo A. F. Gregory T. R. (2014). The case for junk DNA. *PLoS Genet.* 10:e1004351 10.1371/journal.pgen.1004351 PMC4014423 24809441

[B101] Pearson C. E. (2011). Repeat associated non-ATG translation initiation: one DNA, two transcripts, seven reading frames, potentially nine toxic entities! *PLoS Genet.* 7:e1002018 10.1371/journal.pgen.1002018 PMC3053344 21423665

[B102] Persengiev S. Kondova I. Otting N. Koeppen A. H. Bontrop R. E. (2011). Genome-wide analysis of miRNA expression reveals a potential role for miR-144 in brain aging and spinocerebellar ataxia pathogenesis. *Neurobiol. Aging* 32 2316 e2317–2316 e2327. 10.1016/j.neurobiolaging.2010.03.014 20451302

[B103] Prasanth K. V. Prasanth S. G. Xuan Z. Hearn S. Freier S. M. Bennett C. F. (2005). Regulating gene expression through RNA nuclear retention. *Cell* 123 249–263 10.1016/j.cell.2005.08.033 16239143

[B104] Pulst S. M. Nechiporuk A. Nechiporuk T. Gispert S. Chen X. N. Lopes-Cendes I. (1996). Moderate expansion of a normally biallelic trinucleotide repeat in spinocerebellar ataxia type 2. *Nat. Genet.* 14 269–276 10.1038/ng1196-269 8896555

[B105] Robberecht W. Philips T. (2013). The changing scene of amyotrophic lateral sclerosis. *Nat. Rev. Neurosci.* 14 248–264 10.1038/nrn3430 23463272

[B106] Roshan R. Shridhar S. Sarangdhar M. A. Banik A. Chawla M. Garg M. (2014). Brain-specific knockdown of miR-29 results in neuronal cell death and ataxia in mice. *RNA* 20 1287–1297 10.1261/rna.044008.113 24958907 PMC4105753

[B107] Ross C. A. Becher M. W. Colomer V. Engelender S. Wood J. D. Sharp A. H. (1997). Huntington’s disease and dentatorubral-pallidoluysian atrophy: proteins, pathogenesis and pathology. *Brain Pathol.* 7 1003–1016 10.1111/j.1750-3639.1997.tb00898.x 9217980 PMC8098431

[B108] Ross O. A. Rutherford N. J. Baker M. Soto-Ortolaza A. I. Carrasquillo M. M. Dejesus-Hernandez M. (2011). Ataxin-2 repeat-length variation and neurodegeneration. *Hum. Mol. Genet.* 20 3207–3212 10.1093/Hmg/Ddr227 21610160 PMC3140823

[B109] Sahoo T. Del Gaudio D. German J. R. Shinawi M. Peters S. U. Person R. E. (2008). Prader-Willi phenotype caused by paternal deficiency for the HBII-85 C/D box small nucleolar RNA cluster. *Nat. Genet.* 40 719–721 10.1038/ng.158 18500341 PMC2705197

[B110] Salvi J. S. Chan J. N. Y. Szafranski K. Liu T. T. Wu J. D. Olsen J. B. (2014). Roles for Pbp1 and Caloric Restriction in genome and lifespan maintenance via suppression of RNA-DNA Hybrids. *Dev. Cell* 30 177–191 10.1016/j.devcel.2014.05.013 25073155

[B111] Salvi J. S. Mekhail K. (2015). R-loops highlight the nucleus in ALS. *Nucleus* 10.1080/19491034.2015.1004952 [Epub ahead of print]. PMC4615755 25587791

[B112] Schaefer A. O’carroll D. Tan C. L. Hillman D. Sugimori M. Llinas R. (2007). Cerebellar neurodegeneration in the absence of microRNAs. *J. Exp. Med.* 204 1553–1558 10.1084/jem.20070823 17606634 PMC2118654

[B113] Schaffer A. E. Eggens V. R. Caglayan A. O. Reuter M. S. Scott E. Coufal N. G. (2014). CLP1 founder mutation links tRNA splicing and maturation to cerebellar development and neurodegeneration. *Cell* 157 651–663 10.1016/j.cell.2014.03.049 24766810 PMC4128918

[B114] Schwer B. Schumacher B. Lombard D. B. Xiao C. Y. Kurtev M. V. Gao J. (2010). Neural sirtuin 6 (Sirt6) ablation attenuates somatic growth and causes obesity. *Proc. Natl. Acad. Sci. U.S.A.* 107 21790–21794 10.1073/pnas.1016306107 21098266 PMC3003110

[B115] Seng S. Avraham H. K. Jiang S. Venkatesh S. Avraham S. (2006). KLHL1/MRP2 mediates neurite outgrowth in a glycogen synthase kinase 3beta-dependent manner. *Mol. Cell. Biol.* 26 8371–8384 10.1128/MCB.02167-5 16982692 PMC1636797

[B116] Sheinerman K. S. Tsivinsky V. G. Abdullah L. Crawford F. Umansky S. R. (2013). Plasma microRNA biomarkers for detection of mild cognitive impairment: biomarker validation study. *Aging (Albany, NY)* 5 925–938. 24368295 10.18632/aging.100624 PMC3883708

[B117] Shelkovnikova T. A. Robinson H. K. Troakes C. Ninkina N. Buchman V. L. (2014). Compromised paraspeckle formation as a pathogenic factor in FUSopathies. *Hum. Mol. Genet.* 23 2298–2312 10.1093/hmg/ddt622 24334610 PMC3976330

[B118] Shin D. Shin J. Y. Mcmanus M. T. Ptacek L. J. Fu Y. H. (2009). Dicer ablation in oligodendrocytes provokes neuronal impairment in mice. *Ann. Neurol.* 66 843–857 10.1002/ana.21927 20035504 PMC2885004

[B119] Shindler K. S. Ventura E. Dutt M. Elliott P. Fitzgerald D. C. Rostami A. (2010). Oral resveratrol reduces neuronal damage in a model of multiple sclerosis. *J. Neuroophthalmol.* 30 328–339 10.1097/Wno.0b013e3181f7f833 21107122 PMC3312784

[B120] Skourti-Stathaki K. Proudfoot N. J. (2014). A double-edged sword: r loops as threats to genome integrity and powerful regulators of gene expression. *Genes Dev.* 28 1384–1396 10.1101/gad.242990.114 24990962 PMC4083084

[B121] Skourti-Stathaki K. Proudfoot N. J. Gromak N. (2011). Human senataxin resolves RNA/DNA hybrids formed at transcriptional pause sites to promote Xrn2-dependent termination. *Mol. Cell* 42 794–805 10.1016/j.molcel.2011.04.026 21700224 PMC3145960

[B122] Sun Q. W. Csorba T. Skourti-Stathaki K. Proudfoot N. J. Dean C. (2013). R-Loop stabilization represses antisense transcription at the *Arabidopsis* FLC locus. *Science* 340 619–621 10.1126/science.1234848 23641115 PMC5144995

[B123] Szafranski K. Mekhail K. (2014). The fine line between lifespan extension and shortening in response to caloric restriction. *Nucleus* 5 56–65 10.4161/nucl.27929 24637399 PMC4028356

[B124] Takahama K. Takada A. Tada S. Shimizu M. Sayama K. Kurokawa R. (2013). Regulation of telomere length by G-quadruplex telomere DNA- and TERRA-binding protein TLS/FUS. *Chem. Biol.* 20 341–350 10.1016/j.chembiol.2013.02.013 23521792

[B125] Tao J. Wu H. Lin Q. Wei W. Lu X. H. Cantle J. P. (2011). Deletion of astroglial dicer causes non-cell-autonomous neuronal dysfunction and degeneration. *J. Neurosci.* 31 8306–8319 10.1523/JNEUROSCI.0567-11.2011 21632951 PMC3500097

[B126] Todd P. K. Oh S. Y. Krans A. He F. Sellier C. Frazer M. (2013). CGG repeat-associated translation mediates neurodegeneration in fragile X tremor ataxia syndrome. *Neuron* 78 440–455 10.1016/j.neuron.2013.03.026 23602499 PMC3831531

[B127] Tsoi H. Chan H. Y. (2013). Expression of expanded CAG transcripts triggers nucleolar stress in Huntington’s disease. *Cerebellum* 12 310–312 10.1007/s12311-012-0447-6 23315009

[B128] Tsoi H. Lau T. C. Tsang S. Y. Lau K. F. Chan H. Y. (2012). CAG expansion induces nucleolar stress in polyglutamine diseases. *Proc. Natl. Acad. Sci. U.S.A.* 109 13428–13433 10.1073/pnas.1204089109 22847428 PMC3421186

[B129] Vance C. Rogelj B. Hortobagyi T. De Vos K. J. Nishimura A. L. Sreedharan J. (2009). Mutations in FUS, an RNA processing protein, cause familial amyotrophic lateral sclerosis type 6. *Science* 323 1208–1211 10.1126/science.1165942 19251628 PMC4516382

[B130] Wahba L. Amon J. D. Koshland D. Vuica-Ross M. (2011). RNase H and multiple RNA biogenesis factors cooperate to prevent RNA:DNA hybrids from generating genome instability. *Mol. Cell.* 44 978-988 10.1016/j.molcel.2011.10.017 PMC3271842 22195970

[B131] Wang L. C. Chen K. Y. Pan H. Wu C. C. Chen P. H. Liao Y. T. (2011a). Muscleblind participates in RNA toxicity of expanded CAG and CUG repeats in *Caenorhabditis elegans*. *Cell Mol. Life Sci.* 68 1255–1267 10.1007/s00018-010-0522-4 20848157 PMC11114631

[B132] Wang W. X. Huang Q. Hu Y. Stromberg A. J. Nelson P. T. (2011b). Patterns of microRNA expression in normal and early Alzheimer’s disease human temporal cortex: white matter versus gray matter. *Acta Neuropathol.* 121 193–205 10.1007/s00401-010-0756-0 20936480 PMC3073518

[B133] Wang X. Liu P. Zhu H. Xu Y. Ma C. Dai X. (2009). miR-34a, a microRNA up-regulated in a double transgenic mouse model of Alzheimer’s disease, inhibits bcl2 translation. *Brain Res. Bull.* 80 268–273 10.1016/j.brainresbull.2009.08.006 19683563

[B134] Wirdefeldt K. Adami H. O. Cole P. Trichopoulos D. Mandel J. (2011). Epidemiology and etiology of Parkinson’s disease: a review of the evidence. *Eur. J. Epidemiol.* 26(Suppl. 1), S1–S58 10.1007/s10654-011-9581-6 21626386

[B135] Wood S. H. Craig T. Li Y. Merry B. De Magalhaes J. P. (2013). Whole transcriptome sequencing of the aging rat brain reveals dynamic RNA changes in the dark matter of the genome. *Age* 35 763–776 10.1007/s11357-012-9410-1 22555619 PMC3636386

[B136] Wu P. Zuo X. L. Deng H. L. Liu X. X. Liu L. Ji A. M. (2013). Roles of long noncoding RNAs in brain development, functional diversification and neurodegenerative diseases. *Brain Res. Bull.* 97 69–80 10.1016/j.brainresbull.2013.06.001 23756188

[B137] Xu Z. H. Poidevin M. Li X. K. Li Y. J. Shu L. Q. Nelson D. L. (2013). Expanded GGGGCC repeat RNA associated with amyotrophic lateral sclerosis and frontotemporal dementia causes neurodegeneration. *Proc. Natl. Acad. Sci. U.S.A.* 110 7778–7783 10.1073/pnas.1219643110 23553836 PMC3651485

[B138] Yeo A. J. Becherel O. J. Luff J. E. Cullen J. K. Wongsurawat T. Jenjaroenpoon P. (2014). R-loops in proliferating cells but not in the brain: implications for AOA2 and other autosomal recessive ataxias. *PLoS ONE* 9:e90219 10.1371/journal.pone.0090219 PMC3956458 24637776

[B139] Yuce O. West S. C. (2013). Senataxin, defective in the neurodegenerative disorder Ataxia with oculomotor apraxia 2 lies at the interface of transcription and the DNA damage response. *Mol. Cell. Biol.* 33 406–417 10.1128/Mcb.01195-2 23149945 PMC3554130

[B140] Zu T. Gibbens B. Doty N. S. Gomes-Pereira M. Huguet A. Stone M. D. (2011). Non-ATG-initiated translation directed by microsatellite expansions. *Proc. Natl. Acad. Sci. U.S.A.* 108**,** 260–265 10.1073/pnas.1013343108 21173221 PMC3017129

